# Coffee Leaf Rust (*Hemileia vastatrix*) from the Recent Invasion into Hawaii Shares a Genotypic Relationship with Latin American Populations

**DOI:** 10.3390/jof8020189

**Published:** 2022-02-15

**Authors:** Luis A. Ramírez-Camejo, Lisa M. Keith, Tracie Matsumoto, Lionel Sugiyama, Mach Fukada, Mia Brann, Ariana Moffitt, Jingyu Liu, M. Catherine Aime

**Affiliations:** 1 Department of Botany and Plant Pathology, Purdue University, West Lafayette, IN 47907, USA; ramirezcamejo@gmail.com (L.A.R.-C.); mbrann@purdue.edu (M.B.); amoffit@purdue.edu (A.M.); liu1643@purdue.edu (J.L.); 2 Centro de Biodiversidad y Descubrimiento de Drogas, Instituto de Investigaciones Científicas y Servicios de Alta Tecnología (INDICASAT-AIP), City of Knowledge, Panama City P.O. Box 0843-01103, Panama; 3 Coiba Scientific Station (COIBA AIP), City of Knowledge, Clayton, Panama City P.O. Box 0843-01853, Panama; 4 Daniel K. Inouye U.S. Pacific Basin Agricultural Research Center, Agricultural Research Service, USDA, Hilo, HI 96720, USA; lisa.keith@usda.gov (L.M.K.); tracie.matsumoto@usda.gov (T.M.); lionel.sugiyama@usda.gov (L.S.); 5 Hawaii Department of Agriculture, Plant Pest Control Branch, Kahului, HI 96732, USA; mach.t.fukada@hawaii.gov

**Keywords:** invasive diseases, plant pathogens, Pucciniales, rust fungi, tropical fungi

## Abstract

Hawaii has long been one of the last coffee-producing regions of the world free of coffee leaf rust (CLR) disease, which is caused by the biotrophic fungus *Hemileia vastatrix*. However, CLR was detected in coffee farms and feral coffee on the island of Maui in February 2020 and subsequently on other islands of the Hawaiian archipelago. The source of the outbreak in Hawaii is not known, and CLR could have entered Hawaii from more than 50 coffee-producing nations that harbor the pathogen. To determine the source(s) of the Hawaii inoculum, we analyzed a set of eleven simple sequence repeat markers (SSRs) generated from Hawaii isolates within a dataset of 434 CLR isolates collected from 17 countries spanning both old and new world populations, and then conducted a minimum spanning network (MSN) analysis to trace the most likely pathway that *H. vastatrix* could have taken to Hawaii. Forty-two multilocus genotypes (MLGs) of *H. vastatrix* were found in the global dataset, with all isolates from Hawaii assignable to MLG 10 or derived from it. MLG 10 is widespread in Central America and Jamaica, making this region the most probable source of inoculum for the outbreak in Hawaii. An examination of global weather patterns during the months preceding the introduction of CLR makes it unlikely that the pathogen was windborne to the islands. Likely scenarios for the introduction of CLR to Hawaii are the accidental introduction of spores or infected plant material by travelers or seasonal workers, or improperly fumigated coffee shipments originating from Central America or the Caribbean islands.

## 1. Introduction

The coffee leaf rust (CLR) fungus, *Hemileia vastatrix* Berk. & Broome (Pucciniales, Basidiomycota), is the most destructive and economically important disease of coffee (*Coffea arabica* and *C. canephora*) [1,2,3,4,5]. Although CLR disease is not generally fatal to coffee plants, it can affect the growth and fruiting ability [6], causing annual losses exceeding USD two billion [3]. CLR is notorious for its ability to break down host resistance [7,8,9], which can result in huge socio-economic impacts in affected regions [10]. 

Historically, the intensification of coffee production and changing crop management patterns were major influences on the development of CLR epidemics of cultivated coffee [11,12]. For instance, in Ethiopia, where *C. arabica* likely originated, *H. vastatrix* does not cause serious epidemics, even where coffee is cropped, probably due to interactions of CLR with other organisms (e.g., mycoparasitism) or the structure of the forest ecosystems where *C. arabica* still grows wild [12]. The first known epidemics of CLR date to 1869 in Ceylon (now Sri Lanka) when a farmer in Madulsima noticed the first tell-tale orange spots on coffee leaves. These rapidly dispersed to all coffee-growing plantations in the country [12]. Between 1869 and 1985, the disease also spread to every major coffee-growing region of the world, with the exception of Hawaii [3,12,13,14]. 

Hawaii is the largest producer of coffee in the United States. In 2020, Hawaiian coffee production reached 4.3 million lbs produced in 6.8 thousand harvested acres, and was valued at USD 48.38 million (https://www.nass.usda.gov (accessed on 15 January 2022)). The majority of Hawaiian coffee comes from Kauai, Kona, Maui, and Molokai. Kona typica (*C. arabica* cv. typica) is the prominent variety grown in Hawaii due to its quality and flavor, but other varieties are cultivated, such as Caturra, Catuai, Bourbon, SL-28, and Geisha (www.hdoa.hawaii.gov (accessed on 15 January 2022)).

**Figure 1:**
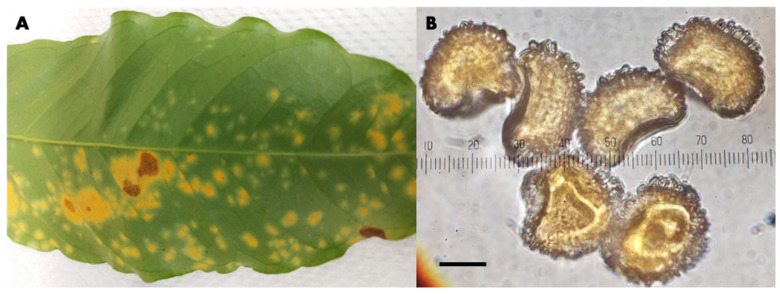
*Hemileia vastatrix* in Kona, Hawaii. (**A**) Infected coffee leaf; (**B**) characteristic hump-backed urediniospores; bar = 10 µm.

At least 90 species of rust fungi have been described from endemic and native hosts in the Hawaiian Islands, with a large percentage apparently introduced [15,16,17]. Nonetheless, for 200 years, Hawaii remained one of the last coffee-growing regions free of CLR [12,14], thanks in part to its geographical isolation and strict plant importation regulations. The absence of CLR in Hawaii changed when spores of *H. vastatrix* were detected in spore traps in Hanaula, Maui, in February 2020 (Keith, pers. obs.), prior to the first discovery of CLR in October 2020 by the Hawaii Department of Agriculture (HDOA) on Haiku, Maui [18]. By July 2021, the disease was confirmed on all major islands of Hawaii where coffee is commercially grown, including Hawaii Island, Lanai, Oahu, Molokai, and Kauai [19] (Figure 1). Pruning methods and spraying approved fungicides were recommended to decrease the infection of CLR in non-resistant coffee varieties in Hawaii [20,21].

Many invasive species are difficult to eradicate, causing incalculable damage to natural ecosystems and significant economic losses to agriculture and forestry [22]. The introduction of invasive species and the costs associated with their management have increased due to the expansion of transportation networks and global trade [23] or due to natural and anthropogenic factors. Therefore, understanding the routes by which *H. vastatrix* travels and applying the political will to reduce these pathways may make it possible to reduce the rate of invasion and to mitigate the extent of damage in Hawaii and elsewhere.

The identification of physiological races through pathogenicity on coffee cultivars has been the primary tool for typing CLR [8,24]. However, recently, molecular markers have emerged as a powerful tool for describing the genotypic diversity and population structure of this fungus [25,26,27,28,29,30]. In this study, we use a set of 11 previously [29] and newly designed simple sequence repeats (SSRs) specific to CLR to genotype 434 *H. vastatrix* isolates from Hawaii and around the world. We conducted a minimum spanning network (MSN) analysis to gain insight into the probable source of inoculum of *H. vastatrix* for Hawaii. The SSR markers were chosen to explore the genetic profile because they are broadly distributed throughout the genome of eukaryotes, are highly polymorphic, and can be located in both protein-coding and noncoding regions [31,32]. Additionally, we examined wind patterns over the Hawaiian Islands prior to the first detection of *H. vastatrix* and established the CLR disease to test the hypothesis that *H. vastatrix* was introduced via wind. 

## 2. Materials and Methods

### Hemileia vastatrix Specimens

A total of 434 specimens of *H. vastatrix* were assembled from new collections and historical herbarium material from 18 countries spanning the range of CLR (Table S1). All specimens were collected on *Coffea arabica* from commercial coffee plantations and farms, except seven specimens collected from *C. canephora* in Cameroon, Thailand, and Jamaica, and one specimen from *C. liberica* from Indonesia. Coffee leaves were dried in a plant press and stored at the Arthur Fungarium (PUR) at Purdue University.

The isolates from Hawaii were collected from CLR-infected leaves on Maui from the site where the initial observance of CLR was recorded and from feral coffee along the roadside from the north side of Maui. Isolates from Hawaii Island were collected from infected leaves from feral and coffee farm locations in Kona. Spore samples were collected in gelatin capsules (Fagron Inc., St. Paul, MN, USA) using a G-R Electric Manufacturing Portable Vacuum Pump with a mini cyclone spore adapter (Tallgrass Solutions, Manhattan, KS, USA).

## 3. PCR, Sequencing, and SSRs 

Spores for each isolate were excised from one to three adjoining sori on a single leaf, and genomic DNA was extracted using the DNeasy PowerPlant Pro Kit (Qiagen, Hilden, Germany) or the PowerPlant Pro DNA isolation Kit (MoBio, Carlsbad, CA, USA). Isolates from spore capsules were similarly extracted, except that a small scoop of spores was used in lieu of excised sori. To confirm that the isolates from Hawaii were *H. vastatrix*, the nuclear large subunit rDNA was amplified following protocols of Aime (2006) and Aime et al. (2018).

SSRs followed the instructions of a modified M13-tailed primer method [33], as previously described [34,35], utilizing 0.2 ng/μL of genomic DNA for each amplification. A total of 11 SSR markers were amplified for each isolate: the eight markers described previously [29], and an additional three markers (Table S2) that were screened and identified following the methods described in [29]. Amplifications followed Ramírez-Camejo et al. (2021), with an exception being that an annealing temperature of 57.6 °C was used for the three new markers (Table S2).

After amplification, the reaction products were diluted and mixed with HiDi^TM^ formamide (Thermo Fisher Scientific, Waltham, MA, USA) loading buffer and GeneScan^TM^ 500 LIZ^TM^ dye size standard (Thermo Fisher Scientific). The products were separated by capillary electrophoresis on an ABI 3730XL Genetic Analyzer to generate fragment data at CD Genomics (www.cd-genomics.com (accessed on 15 January 2022). The fragment sizes for each isolate at all 11 loci were determined using Geneious v9.1.8 (Biomatters Ltd., Auckland, New Zealand) [36].

## 4. SSRs and Minimum Spanning Network

The global data matrix based on the eleven SSR markers was transformed to GenAlEx v6.5 format (Table S3) [37,38]. All analyses were performed with R package *Poppr* v2.8.3. To determine the minimum number of loci necessary to discriminate between genotypes, we generated a genotype accumulation curve that was randomly sampled 1000 times for each boxplot [39]. 

The eleven SSR markers were analyzed by the minimum spanning network (MSN) using the interactive tool *imsn()* with 1000 random seeds. The MSN allows for the visualization of genetic relatedness among individual MLGs in the global *H. vastatrix* population [39,40]. We used Nei’s genetic distance, as it has been applied in other analyses of rust fungal populations [41] and our prior work [29]. 

## 5. Wind Pattern Visualizations

Wind pattern visualization was performed using an animated map of global weather conditions on Earth (https://earth.nullschool.net (accessed on 15 January 2022)) in Hawaii. The source of the maps are data made by NASA supercomputers updated every three hours. We use the first day of each month (from February 2019 to February 2020) as points of reference of the wind pattern surrounding Hawaii. 

## 6. Results

The identity of the isolates from Hawaii were confirmed by 28S sequencing, sharing a 100% identity with each other, and with previously published sequences, as belonging to *H. vastatrix* [31,42] (GenBank accession number OM487037). The number of SSR loci analyzed was sufficient to observe the total number of MLGs (Figure S1).

**Figure 2:**
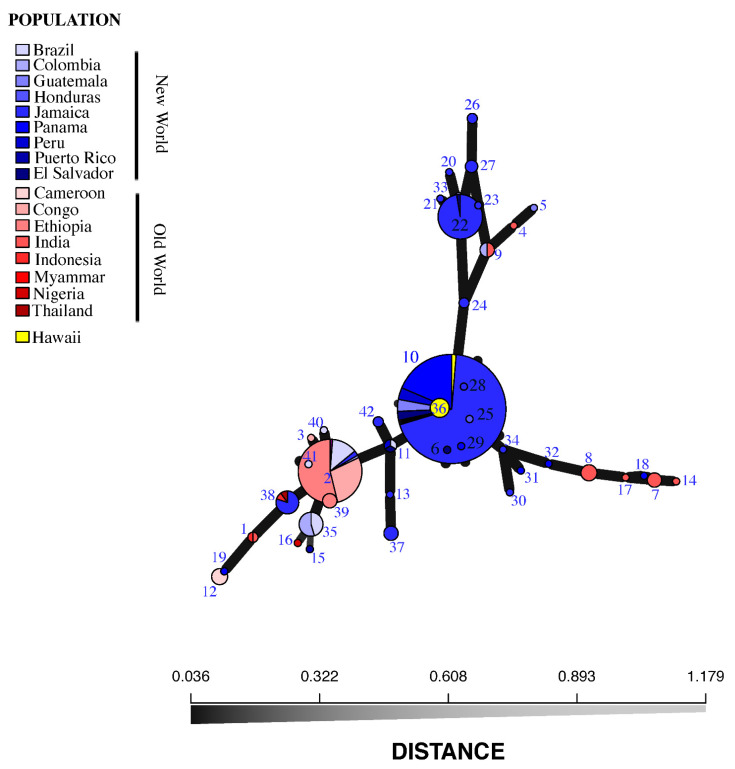
Minimum spanning network (MSN) of the multilocus genotypes (MLGs) of sampled *Hemileia vastatrix* isolates (*n* = 434). The MSN is based on Nei’s genetic distance. Each node represents a unique MLG. Node size corresponds to the number of individual isolates comprising each MLG; colors correspond to geographic origin of isolates; edge thickness is proportional to Nei’s genetic distance.

A total of 42 MLGs were identified (1–42, Figure 2), distributed among the 434 *H. vastatrix* isolates (Figure 2 and Figure S2). We found unique MLGs that are present in only one country, e.g., Cameroon (MLG 12; *n* = 5), Puerto Rico (MLG 15; *n* = 1), Nigeria (MLG 16; *n* = 1), Honduras (MLG 25; *n* = 1), Colombia (MLG 33; *n* = 1), Hawaii (MLG 36; *n* = 7), India [(MLG 4, 14, 17; *n* = 1 for each MLG) (MLG 7; *n* = 4) (MLG 8; *n* = 5)], Guatemala (MLG 5; *n* = 1), Jamaica [(MLG 37; *n* = 4) (MLG 42, 24, 26; *n* = 2 for each MLG) (MLG 13, 20, 21, 23, 28, 30, 34; *n* = 1 for each MLG) (MLG 27; *n* = 3)], El Salvador (MLG 6; *n* = 1), Panama [(MLG 18, 19, 29, 31, 32; *n* = 1 for each MLG)], Congo (MLG 3; *n* = 1), Ethiopia (MLG 39; *n* = 4), and Brazil (MLG 40, 41; *n* = 1 for each MLG) (Figure 2 and Figure S2). Some MLGs were shared between some but not all countries, e.g., Colombia and Brazil (MLG 35; *n* = 11); El Salvador and Jamaica (MLG 22; *n* = 38); Colombia and India (MLG 9; *n* = 4); Panama, Guatemala, Peru, El Salvador, India, Puerto Rico, Honduras, Jamaica, and Hawaii (MLG 10; *n* = 226); Peru, El Salvador, and Brazil (MLG 11; *n* = 3); Indonesia and India (MLG 1; *n* = 2); Thailand, Indonesia, and Jamaica (MLG 38; *n* = 10); and Ethiopia, Congo, Colombia, Jamaica, Brazil, and Myanmar (MLG 2; *n* = 78) (Figure 2 and Figure S2).

The prevailing wind patterns over Hawaii from February 2019 to February 2020 on the islands were derived from North America, rather than South or Central America, Africa, or Asia (Figure S3).

## 7. Discussion

In this study, we generated eleven SSR markers for the most complete published sampling of *H. vastatrix* specimens, which include new and historic herbarium collections from 18 countries around the world, including all coffee-growing regions. The data were analyzed to determine the potential source of the recent introduction of CLR into Hawaii. A total of 42 MLGs (42 MLG/ *n* = 434, or 0.10) were identified from this sampling, which is similar to that found in our previous study (12 MLG/ *n* = 105, or 0.11), as well as to other rust fungi, such as *Puccinia striiformis* f. sp. *tritici* in the United States (MLGs = 32/*n* = 270, or 0.12) [29,43]. 

The distribution of MLGs shows the presence of several unique MLGs, as well as several that are shared (Figure S2). The specimens from Hawaii were all derived from MLG 10 (Figure 2), which comprises 226 isolates from Panama, Guatemala, El Salvador, Honduras, Puerto Rico, and Jamaica, plus one (of sixteen total) isolate from India, and eight (of nine total) isolates from Peru, all from *C. arabica*, with the exception of one isolate originating from *C. canephora* in Jamaica. The existence of identical genotypes (MLGs) in seven countries of Latin America and the Caribbean with a single isolate from India has been attributed to “founder effects” through old and/or recent epidemic events in uniform and susceptible coffee crops where genotypes are rapidly propagated without losing effective combinations of genes [29,44,45]. More importantly, the identification of MLGs from the predominantly Central American genotype suggests that the initial source of inoculum in Hawaii came from this region. 

There are at least three potential hypotheses for the source of the inoculum that led to the outbreak of CLR in Hawaii [46]. First, *H. vastatrix* was present in Hawaii either on other hosts or in a specific coffee cultivar, and changes in coffee management practices led to an outbreak. This is the least likely explanation, as the other two species of *Coffea* (*C. mariniana* and *C. odorata*) native to Hawaii are not known to act as alternative hosts. Al-though CLR was first discovered on a farm in Haiku, Maui, the majority of the CLR was first discovered in feral or unmanaged fields. This suggests that the nutrition and cultural practices to improve aeration within the canopy may deter initial CLR infection. Since the discovery of the coffee berry borer (CBB) in 2010, stump pruning was recommended to reduce the CBB levels in the field. Until the discovery of CLR in Hawaii in 2020, no major changes in management practices occurred, and it was only after CLR arrived in the State of Hawaii that fungicide applications were recommended to decrease the infection in coffee-growing areas [20,21]. 

Secondly, viable urediniospores were transported to Hawaii by wind. Trade winds are one of the largest and most consistent winds on Earth [47] and are prevalent over the islands of Hawaii throughout the year due to high pressure systems that form in the North Pacific [48]. According to the NOAA’s National Centers for Environmental Information (https://www.ncdc.noaa.gov/stormevents/ (accessed on 12 August 2021)), over 26 hurricanes or their remnants have hit the Hawaiian Islands between 1955–2021, generating swells, heavy precipitation, and power outages, and sometimes damaging crops. For example, in 2014, with an estimated wind intensity of 140 mph, Hurricane Isabelle caused significant losses of over USD 50 million to a variety of crops, including coffee [49]. In 2020, Hurricane Douglas generated swells of 10 to 20 feet along the east-facing shores of Hawaii Island, Maui, Molokai, and Kahoolawe, but there were no reports of serious injuries or property damage.

These tropical cyclones and trade winds could have served as an inoculum source of CLR from Latin America to Hawaii. However, the wind patterns observed over Hawaii prior to CLR discovery originated from North America, where coffee crops are absent (Figure S3), and this pattern remains unchanged. Additionally, prior to 2020, aerobiological sequencing has never detected *H. vastatrix* in over 13 years of sampling over Hawaii Island and Mauna Loa Observatory [50], making an aerial route for the introduction of the pathogen unlikely. Finally, the trajectory of the major meteorological events, such as Hurricane Douglas in 2020, originated primarily from the Pacific Ocean rather than the Americas. This contrasts with the movement of urediniospores of the sugar cane rust *Puccinia melanocephala* to America from Africa due to tropical weather disturbance based on airflow patterns analysis from Cameroon to the Dominican Republic [46]. It has been hypothesized that wind currents may have carried spores of *H. vastatrix* across the Atlantic from Angola to Brazil [51]. However, there is little probability that CLR can effectively travel long distances via spores, because, unlike the urediniospores of *Puccinia* and related species that are heavily melanized, *H. vastatrix* spores are not melanized and likely lose viability due to UV and desiccation during long distance travel outside of the host [52]. Even melanized urediniospores of the wheat rust pathogen *Puccinia* species (*P. striiformis*, *P. recondita*, and *P. graminis*) were sensitive to the UV radiation of various wavelengths and, in particular, to wavelengths present in sunlight [53]. This implies that even if *H. vastatrix* spores were windborne to Hawaii, the probability of viable germination on a susceptible coffee plant is minimal. This is supported by the absence of genotypes from Africa into Hawaiian specimens of *H. vastatrix*, suggesting that their urediniospores have not come from that coffee-growing region of the world.

Thirdly, *H. vastatrix* was introduced into Hawaii anthropogenically through the transport of infected plant material or spores. Coffee was originally brought to Hawaii in 1817, but the plantings did not succeed [54]. In 1825, plants were brought in from Brazil and the first commercial coffee plantation was started in Koloa, Kauai, 11 years later [55]. According to the Plant Industry Division of Hawaii (https://hdoa.Hawaii.gov/pi/ (accessed on 15 January 2021)), to avoid the entry of pests that would damage the industry of coffee, the introduction of any foreign coffee plant (plant parts, seeds, plastic bags) into Hawaii has been prohibited since 1888. Plants can only be directly imported to Hawaii from the continental U.S. and is subject to a one year quarantine. However, under the Code of Federal Regulations, exceptions to coffee plant importations were given with the correspondent permits [56,57]. Basically, all plants imported require importation permits, a U.S. Customs declaration, and examination by Plant Protection and Quarantine Service inspectors. Despite strict regulation rules, CLR may bypass these regulations. For example; approximately 500 coffee-growing kits containing several African coffee seeds were imported from USA to Hawaii in 2017 without any permits, and many of them were sold on Kauai and Oahu [58]. In 2005, the guava/eucalyptus rust fungus *Austropuccinia psidii* (formerly known as *Puccinia psidii*) spread from its origin in Latin America to Hawaii [59,60]. Although there are many pathways by which *A. psidii* could reach Hawaii from infected areas, the most likely pathway of entry is by the nursery stock or foliage of the plant family Myrtaceae [59]. This is based on several intercepted myrtle samples from California shippers containing rust by Maui HDOA inspectors from 2006 to 2007. In 2010, the coffee berry borer *Hypothenemus hampei* was detected in coffee plantations of Hawaii Island. Phylogenetic and haplotype network analysis suggested that the most likely route of invasion was from Kenya to Uganda to Latin America to Hawaii via accidental transport by farm workers, growers, or outside travelers [22]. Due to the fact that the incubation period for *H. vastatrix* in coffee plants is approximately 4 to 7 weeks post initial infection before visible signs of the disease are apparent [61], asymptomatic but infected coffee plants with CLR could have gone unnoticed during inspections in Hawaii. 

Coffee farms employ migrant workers from other coffee-growing countries to help during the coffee harvest season each year, which may inadvertently transport spores on clothing and other materials (Matsumoto, pers. obs.). As was the case for *A. psidii*, *H. hampei*, and other introduced pests in Hawaii [62], it is possible that CLR in Hawaii was accidentally transported by farm workers or local and foreign travelers from Central America or the Caribbean who carried infected seedlings plant material or spores in their clothing or luggage. This hypothesis is based on the MSN data, where three specimens of the Hawaii MLGs (MLG 10) shared identical genotypes with 222 specimens found in El Salvador, Guatemala, Honduras, Jamaica, Panama, Peru, and Puerto Rico (Figure 2), rather than with MLGs originating from old world populations. MLG 10 almost exclusively comprises CLR collected following the 2012 epidemic in Central America known as the “big rust” [10], and is the largest MLG in our dataset.

In addition to MLG 10, several isolates from Hawaii comprised a new genotype, MLG 36, that is derived from MLG 10 (Figure 2). Two likely hypotheses can explain this pattern: (1) CLR has been present in Hawaii long enough for a new MLG to have evolved from a founder population of MLG 10; or (2) MLG 36 evolved from a founder population in Central America and more than one introduction of CLR was made into Hawaii. Our data are consistent from other infected coffee plantations with CLR worldwide and it is necessary to explore the possibility of having a double introduction of CLR into Hawaii.

In summary, before 2020, coffee grown in Hawaii was free of CLR due to its geographical isolation and plant regulations. Our data are consistent with a hypothesis that the CLR outbreak in Hawaii originated from introduced infected coffee plant material or was accidentally brought in on the clothing of people traveling from Central America or the Caribbean (either vacationers returning home or migrant workers) and belongs to the same genotype responsible for the “big rust” epidemic of 2012 in the Americas. We cannot discard the possibility that other coffee-growing regions not sampled here (for example, Mexico) can be a candidate for the origin of the outbreak, assuming that they are indistinguishable genetically with genotypes found in Hawaii.

## Supplementary Materials

The following supporting information can be downloaded at: https://www.mdpi.com/article/10.3390/jof8020189/s1. Table S1. *Hemileia vastatrix* isolates examined in this study. Table S2. Three newly generated SSRs used in this study. Table S3. Input file of *Hemileia vastatrix* data. Figure S1. Genotype accumulation curve for eleven SSR markers characterized in 434 *Hemileia vastatrix* specimens from global population. Figure S2. Distribution of genotypic diversity in 434 specimens of *Hemileia vastatrix*. Figure S3. Global wind pattern during the first day each from February 2019 to October 2020.
