# Supplementary material for: Coffee Leaf Rust (Hemileia vastatrix) from the Recent Invasion into Hawaii Shares a Genotypic Relationship with Latin American Populations
Source: J Fungi (Basel). 2022 Feb 15;8(2):189. doi: 10.3390/jof8020189 (PMC8877902; doi:10.3390/jof8020189)
Supplement: Supplementary file 1 [file jof-08-00189-s001.zip › jof-1586548-supplementary.pdf]

Supplemental Material

Table S1. *Hemileia vastatrix* isolates examined in this study.

Specimens ID/Herbarium voucher	Host	Variety	Country	Location	Year of Collection	Altitude (m)	Latitude	Longitude	MLG
A	*Coffea arabica*	---	Ethiopia	SNNPR	2017	1714	7.28	36.23	2
AB	*Coffea arabica*	---	Ethiopia	SNNPR	2017	1815	7.28	36.23	2
AD	*Coffea arabica*	---	Ethiopia	SNNPR	2017	1805	7.28	36.24	2
AE	*Coffea arabica*	---	Ethiopia	SNNPR	2017	1806	7.3	36.2	2
AG	*Coffea arabica*	---	Ethiopia	SNNPR	2017	1720	7.3	36.2	39
AH	*Coffea arabica*	---	Ethiopia	SNNPR	2017	1726	7.3	36.2	2
AJ	*Coffea arabica*	---	Ethiopia	SNNPR	2017	1720	7.3	36.2	2
AK	*Coffea arabica*	---	Ethiopia	SNNPR	2017	1717	7.28	36.23	2
AL	*Coffea arabica*	---	Ethiopia	Oromia	2017	1567	7.85	36.66	2
AM	*Coffea arabica*	---	Ethiopia	Oromia	2017	1845	9.2	35.8	2
AO	*Coffea arabica*	---	Ethiopia	Oromia	2017	1843	9.17	35.84	2
AR	*Coffea arabica*	---	Ethiopia	Oromia	2017	1850	9.2	35.8	2
AS	*Coffea arabica*	---	Ethiopia	Oromia	2017	1843	9.2	35.8	2
AT	*Coffea arabica*	---	Ethiopia	Oromia	2017	1845	9.17	35.83	2
AV	*Coffea arabica*	---	Ethiopia	Oromia	2017	1715	7.67	36.83	2
AW	*Coffea arabica*	---	Ethiopia	Oromia	2017	1718	7.67	36.83	39
AY	*Coffea arabica*	---	Ethiopia	Oromia	2017	1721	7.7	36.8	2
AZ	*Coffea arabica*	---	Ethiopia	Oromia	2017	1718	7.7	36.8	39
BA	*Coffea arabica*	---	Ethiopia	Oromia	2017	1753	7.7	36.8	2
BB	*Coffea arabica*	---	Ethiopia	Oromia	2017	1757	7.7	36.8	2
BC	*Coffea arabica*	---	Ethiopia	Oromia	2017	1752	7.67	36.84	2
BD	*Coffea arabica*	---	Ethiopia	Oromia	2017	1757	7.67	36.83	2

BE	*Coffea arabica*	---	Ethiopia	Oromia	2017	1753	7.67	36.83	2
BI	*Coffea arabica*	---	Ethiopia	Oromia	2017	1759	7.67	36.83	2
C	*Coffea arabica*	---	Ethiopia	SNNPR	2017	1529	6.4	38.3	2
CN	*Coffea arabica*	---	Ethiopia	SNNPR	2017	1260	7.2	35.45	2
CO	*Coffea arabica*	---	Ethiopia	SNNPR	2017	1215	7.2	35.45	2
CQ	*Coffea arabica*	---	Ethiopia	SNNPR	2017	1260	7.2	35.5	39
CS	*Coffea arabica*	---	Ethiopia	SNNPR	2017	1240	7.2	35.45	2
CU	*Coffea arabica*	---	Ethiopia	SNNPR	2017	1260	7.2	35.45	2
D	*Coffea arabica*	---	Ethiopia	SNNPR	2017	1531	6.4	38.3	2
N	*Coffea arabica*	---	Ethiopia	SNNPR	2017	1531	6.42	38.32	2
O	*Coffea arabica*	---	Ethiopia	SNNPR	2017	1543	6.4	38.3	2
Q	*Coffea arabica*	---	Ethiopia	SNNPR	2017	1528	6.42	38.32	2
T	*Coffea arabica*	---	Ethiopia	SNNPR	2017	1514	6.4	38.3	2
U	*Coffea arabica*	---	Ethiopia	SNNPR	2017	1240	7.2	35.45	2
MCA8388_1	*Coffea arabica*	---	Ethiopia	SNPR	2019	1558	7.17897	36.27075	2
MCA8388_2	*Coffea arabica*	---	Ethiopia	SNPR	2019	1558	7.17897	36.27075	2
MCA8388_3	*Coffea arabica*	---	Ethiopia	SNPR	2019	1558	7.17897	36.27075	2
MCA8388_4	*Coffea arabica*	---	Ethiopia	SNPR	2019	1558	7.17897	36.27075	2
MCA8388_5	*Coffea arabica*	---	Ethiopia	SNPR	2019	1558	7.17897	36.27075	2
MCA8392_1	*Coffea arabica*	---	Ethiopia	SNPR	2019	1755	7.2813	36.19387	2
MCA8392_2	*Coffea arabica*	---	Ethiopia	SNPR	2019	1755	7.2813	36.19387	2
MCA8392_3	*Coffea arabica*	---	Ethiopia	SNPR	2019	1755	7.2813	36.19387	2
MCA8392_4	*Coffea arabica*	---	Ethiopia	SNPR	2019	1755	7.2813	36.19387	2
MCA8392_5	*Coffea arabica*	---	Ethiopia	SNPR	2019	1755	7.2813	36.19387	2
RAK420/PUR054576	*Coffea canephora*	Robusta SG2	Cameroon	Somalomo	2018	659	3.38114	12.73431	12
RAK421	*Coffea canephora*	Robusta SG2	Cameroon	Somalomo	2018	659	3.38114	12.73431	12
RAK422/PUR054577	*Coffea canephora*	Robusta SG2	Cameroon	Somalomo	2018	659	3.38114	12.73431	12
RAK422/PUR054578	*Coffea canephora*	Robusta SG2	Cameroon	Somalomo	2018	659	3.38114	12.73431	12

MCA7070/PUR045847	*Coffea canephora*	Robusta SG2	Cameroon	Somalomo	2016	659	3.38114	12.73431	12
PHV001/PUR049703	*Coffea arabica*	Catuai	Panama	Chiriqui	2016	1172	8.74319	-82.46008	10
PHV006/PUR053038	*Coffea arabica*	Typica	Panama	Chiriqui	2015	1448	8.87611	-82.76275	10
PHV008/PUR053039	*Coffea arabica*	Catuai	Panama	Chiriqui	2015	1448	8.87992	-82.76008	10
PHV009/PUR053040	*Coffea arabica*	Catuai	Panama	Chiriqui	2015	1448	8.87992	-82.76008	10
PHV011/PUR053041	*Coffea arabica*	Geisha	Panama	Chiriqui	2015	1449	8.87978	-82.75944	10
PHV013/PUR053043	*Coffea arabica*	Typica	Panama	Chiriqui	2016	1284	8.86064	-82.77456	10
PHV014/PUR053044	*Coffea arabica*	Typica	Panama	Chiriqui	2016	1448	8.87611	-82.76275	10
PHV015	*Coffea arabica*	Typica	Panama	Chiriqui	2016	1448	8.87611	-82.76275	10
PHV016	*Coffea arabica*	Typica	Panama	Chiriqui	2016	1448	8.87611	-82.76275	10
PHV021	*Coffea arabica*	Geisha	Panama	Chiriqui	2016	1449	8.87978	-82.75944	10
PHV022	*Coffea arabica*	Catuai	Panama	Chiriqui	2016	1448	8.87992	-82.76008	10
PHV023	*Coffea arabica*	Catuai	Panama	Chiriqui	2016	1172	8.74319	-82.46008	10
PHV030	*Coffea arabica*	Catuai	Panama	Chiriqui	2016	1172	8.74319	-82.46008	10
PHV034	*Coffea arabica*	Typica	Panama	Ngäbe-Buglé Comarca	2016	1357	8.48344	-81.76786	10
PHV036	*Coffea arabica*	---	Panama	Ngäbe-Buglé Comarca	2016	1357	8.48344	-81.76786	10
PHV037	*Coffea arabica*	---	Panama	Ngäbe-Buglé Comarca	2016	1357	8.48344	-81.76786	10
PHV039	*Coffea arabica*	Typica	Panama	Chiriqui	2016	1284	8.86064	-82.77456	10
PHV040	*Coffea arabica*	Typica	Panama	Chiriqui	2016	1284	8.86064	-82.77456	10
PHV043	*Coffea arabica*	Geisha	Panama	Chiriqui	2016	1449	8.87978	-82.75944	10
PHV045	*Coffea arabica*	Typica	Panama	Chiriqui	2016	1284	8.86064	-82.77456	10
PHV050	*Coffea arabica*	Catuai	Panama	Chiriqui	2016	1448	8.87992	-82.76008	10
PHV057	*Coffea arabica*	Catuai	Panama	Chiriqui	2016	1172	8.74319	-82.46008	18
PHV058	*Coffea arabica*	Geisha	Panama	Chiriqui	2016	1132	8.742	-82.46008	10
PHV060	*Coffea arabica*	---	Panama	Ngäbe-Buglé Comarca	2016	1357	8.48344	-81.76786	10
PHV061	*Coffea arabica*	---	Panama	Ngäbe-Buglé Comarca	2016	1357	8.48344	-81.76786	10
PHV062	*Coffea arabica*	---	Panama	Ngäbe-Buglé Comarca	2016	1357	8.48344	-81.76786	10
PHV067	*Coffea arabica*	---	Panama	Ngäbe-Buglé Comarca	2016	1357	8.48344	-81.76786	10

PHV068	*Coffea arabica*	---	Panama	Ngäbe-Buglé Comarca	2016	1357	8.48344	-81.76786	32
PHV070	*Coffea arabica*	Geisha	Panama	Chiriqui	2016	1132	8.742	-82.46008	10
PHV071	*Coffea arabica*	Catuai	Panama	Chiriqui	2016	1172	8.74319	-82.46008	10
PHV072	*Coffea arabica*	Geisha	Panama	Chiriqui	2016	1132	8.742	-82.46008	10
PHV073	*Coffea arabica*	Catuai	Panama	Chiriqui	2016	1172	8.74319	-82.46008	10
PHV075	*Coffea arabica*	Catuai	Panama	Chiriqui	2016	1448	8.87611	-82.76275	10
PHV076	*Coffea arabica*	Catuai	Panama	Chiriqui	2016	1448	8.87611	-82.76275	10
PHV077	*Coffea arabica*	Geisha	Panama	Chiriqui	2016	1449	8.87978	-82.75944	10
PHV083	*Coffea arabica*	---	Panama	Ngäbe-Buglé Comarca	2016	1357	8.48344	-81.76786	10
PHV084	*Coffea arabica*	Geisha	Panama	Ngäbe-Buglé Comarca	2016	1357	8.48344	-81.76786	10
PHV090	*Coffea arabica*	Geisha	Panama	Chiriqui	2016	1132	8.742	-82.46008	10
PHV092	*Coffea arabica*	Geisha	Panama	Chiriqui	2016	1132	8.742	-82.46008	10
PHV100	*Coffea arabica*	Catuai	Panama	Ngäbe-Buglé Comarca	2016	1357	8.48344	-81.76786	31
PHV104	*Coffea arabica*	Catuai	Panama	Ngäbe-Buglé Comarca	2016	1357	8.48344	-81.76786	10
PHV108	*Coffea arabica*	Typica	Panama	Chiriqui	2016	1448	8.87611	-82.76275	10
PHV113	*Coffea arabica*	Catuai	Panama	Chiriqui	2016	1448	8.87992	-82.76008	10
PHV114	*Coffea arabica*	Catuai	Panama	Chiriqui	2016	1448	8.87992	-82.76008	19
PHV145/PUR053035	*Coffea arabica*	Geisha	Panama	Chiriqui	2016	1132	8.742	-82.46008	10
PHV146	*Coffea arabica*	Catuai	Panama	Chiriqui	2016	1172	8.74319	-82.46008	29
PHV147/PUR053037	*Coffea arabica*	Geisha	Panama	Chiriqui	2016	1132	8.742	-82.46008	10
WCR5	*Coffea arabica*	K7	DRC	Yangambi	2018	423	0.76243	24.46293	2
WCR6	*Coffea arabica*	K7	DRC	Yangambi	2018	423	0.76243	24.46293	2
WCR7	*Coffea arabica*	K7	DRC	Yangambi	2018	423	0.76243	24.46293	2
WCR8	*Coffea arabica*	K7	DRC	Yangambi	2018	423	0.76243	24.46293	2
WCR9	*Coffea arabica*	K7	DRC	Yangambi	2018	423	0.76243	24.46293	2
WCR10	*Coffea arabica*	K7	DRC	Yangambi	2018	423	0.76243	24.46293	2
WCR12	*Coffea arabica*	SL28	DRC	Yangambi	2018	423	0.76243	24.46293	2
WCR13	*Coffea arabica*	SL28	DRC	Yangambi	2018	423	0.76243	24.46293	2

WCR15	*Coffea arabica*	SL28	DRC	Yangambi	2018	423	0.76243	24.46293	2
WCR16	*Coffea arabica*	SL28	DRC	Yangambi	2018	423	0.76243	24.46293	2
WCR17	*Coffea arabica*	SL28	DRC	Yangambi	2018	423	0.76243	24.46293	2
WCR18	*Coffea arabica*	SL28	DRC	Yangambi	2018	423	0.76243	24.46293	2
WCR19	*Coffea arabica*	SL28	DRC	Yangambi	2018	423	0.76243	24.46293	2
WCR20	*Coffea arabica*	SL28	DRC	Yangambi	2018	423	0.76243	24.46293	2
WCR21	*Coffea arabica*	K7	DRC	Yangambi	2018	423	0.76243	24.46293	2
WCR22	*Coffea arabica*	K7	DRC	Yangambi	2018	423	0.76243	24.46293	3
WCR24	*Coffea arabica*	K7	DRC	Yangambi	2018	423	0.76243	24.46293	2
WCR25	*Coffea arabica*	K7	DRC	Yangambi	2018	423	0.76243	24.46293	2
WCR26	*Coffea arabica*	K7	DRC	Yangambi	2018	423	0.76243	24.46293	2
WCR27	*Coffea arabica*	K7	DRC	Yangambi	2018	423	0.76243	24.46293	2
WCR28	*Coffea arabica*	K7	DRC	Yangambi	2018	423	0.76243	24.46293	2
WCR29	*Coffea arabica*	K7	DRC	Yangambi	2018	423	0.76243	24.46293	2
WCR30	*Coffea arabica*	K7	DRC	Yangambi	2018	423	0.76243	24.46293	2
WCR-2	*Coffea arabica*	Catimor	Colombia	Antioquia	2017	2146	7	-75.5	9
CO-1	*Coffea arabica*	Caturra	Colombia	Risaralda	2016	1420	4.96109	-75.6424	35
CO-2	*Coffea arabica*	Caturra	Colombia	Risaralda	2016	1420	4.96109	-75.6424	35
CO-3	*Coffea arabica*	Caturra	Colombia	Risaralda	2016	1420	4.96109	-75.6424	2
CO-4	*Coffea arabica*	Caturra	Colombia	Risaralda	2016	1420	4.96109	-75.6424	33
CO-5	*Coffea arabica*	Caturra	Colombia	Risaralda	2016	1420	4.96109	-75.6424	35
CO-6	*Coffea arabica*	Caturra	Colombia	Risaralda	2016	1420	4.96109	-75.6424	35
CO-8	*Coffea arabica*	Caturra	Colombia	Risaralda	2016	1498	4.93682	-75.65083	35
CO-9	*Coffea arabica*	Caturra	Colombia	Risaralda	2016	1498	4.93682	-75.65083	9
CO-12	*Coffea arabica*	Caturra	Colombia	Risaralda	2016	1498	4.93682	-75.65083	35
GT-3	*Coffea arabica*	---	Guatemala	Acatenango	2016	1625	14.5512	-90.95399	10
GT-4	*Coffea arabica*	---	Guatemala	Acatenango	2016	1625	14.5512	-90.95399	5
GT-5	*Coffea arabica*	---	Guatemala	Acatenango	2016	1625	14.5512	-90.95399	10

GT-6	*Coffea arabica*	---	Guatemala	Acatenango	2016	1625	14.5512	-90.95399	10
GT-7	*Coffea arabica*	---	Guatemala	Acatenango	2016	1625	14.5512	-90.95399	10
GT-8	*Coffea arabica*	---	Guatemala	Acatenango	2016	1625	14.5512	-90.95399	10
GT-9	*Coffea arabica*	---	Guatemala	Acatenango	2016	1625	14.5512	-90.95399	10
GT-11	*Coffea arabica*	---	Guatemala	Acatenango	2016	1625	14.5512	-90.95399	10
GT-12	*Coffea arabica*	---	Guatemala	Acatenango	2016	1625	14.5512	-90.95399	10
MCA6412/PUR045839	*Coffea arabica*	Bourbon	Peru	Piura	2016	282	-5.2643	-79.6788	10
MCA6413/PUR045840	*Coffea arabica*	---	Peru	Piura	2016	282	-5.2643	-79.6788	10
MCA6415/PUR045841	*Coffea arabica*	Typica	Peru	Piura	2016	657	-5.3024	-79.65722	10
MCA6416/PUR045842	*Coffea arabica*	---	Peru	Piura	2016	562	-5.2446	-79.65818	10
MCA6417/PUR045844	*Coffea arabica*	---	Peru	Piura	2016	551	-5.2444	-79.65838	10
MCA6418/PUR045845	*Coffea arabica*	---	Peru	Piura	2016	559	-5.2436	-79.65807	10
MCA6419/PUR045848	*Coffea arabica*	---	Peru	Piura	2016	637	-5.4121	-79.64222	10
PUR045843	*Coffea arabica*	---	Peru	Cajamarca	2016	1016	-5.1968	-78.79373	11
PURN16756	*Coffea arabica*	---	Peru	Piura	2017	506	-5.264	-79.6786	10
WCR ES1	*Coffea arabica*	CR95	El Salvador	Santa Anna	2019	950	13.9172	-89.56932	11
WCR ES2	*Coffea arabica*	T-5296	El Salvador	Santa Anna	2019	950	13.9172	-89.56932	22
WCR ES3	*Coffea arabica*	CR95	El Salvador	Santa Anna	2019	950	13.9172	-89.56932	10
WCR ES4	*Coffea arabica*	CR95	El Salvador	Santa Anna	2019	950	13.9172	-89.56932	10
WCR ES5	*Coffea arabica*	T-5296	El Salvador	Santa Anna	2019	950	13.9172	-89.56932	10
WCR ES6	*Coffea arabica*	Bourbon	El Salvador	Santa Anna	2019	950	13.9172	-89.56932	10
WCR ES7	*Coffea arabica*	Bourbon	El Salvador	Santa Anna	2019	950	13.9172	-89.56932	10
WCR ES8	*Coffea arabica*	CR95	El Salvador	Santa Anna	2019	950	13.9172	-89.56932	10
WCR ES9	*Coffea arabica*	CR95	El Salvador	Santa Anna	2019	950	13.9172	-89.56932	6
WCR_TH3	*Coffea canephora*	Robusta	Thailand	Chumphon	2019	123	10.2655	99.029	38
WCR I1	*Coffea arabica*	AB3	India	Karnataka State	2019	861	13.3739	75.57583	1
WCR I2	*Coffea arabica*	Batian	India	Karnataka State	2019	861	13.3739	75.57583	9
WCR I3	*Coffea arabica*	Col-1	India	Karnataka State	2019	861	13.3739	75.57583	10

WCR I4	*Coffea arabica*	Col-2	India	Karnataka State	2019	861	13.3739	75.57583	17
WCR I5	*Coffea arabica*	Col-3	India	Karnataka State	2019	861	13.3739	75.57583	8
WCR I6	*Coffea arabica*	Col-4	India	Karnataka State	2019	861	13.3739	75.57583	8
WCR I7	*Coffea arabica*	Col-5	India	Karnataka State	2019	861	13.3739	75.57583	8
WCR I8	*Coffea arabica*	Geisha	India	Karnataka State	2019	861	13.3739	75.57583	7
WCR I10	*Coffea arabica*	K7	India	Karnataka State	2019	861	13.3739	75.57583	7
WCR I11	*Coffea arabica*	Lempira	India	Karnataka State	2019	861	13.3739	75.57583	9
WCR I12	*Coffea arabica*	Mundo Novo	India	Karnataka State	2019	861	13.3739	75.57583	7
WCR I13	*Coffea arabica*	Oro Azetica	India	Karnataka State	2019	861	13.3739	75.57583	4
WCR I14	*Coffea arabica*	Prainema	India	Karnataka State	2019	861	13.3739	75.57583	8
WCR I15	*Coffea arabica*	Ruiru 11	India	Karnataka State	2019	861	13.3739	75.57583	8
WCR I16	*Coffea arabica*	SL28	India	Karnataka State	2019	861	13.3739	75.57583	7
WCR I17	*Coffea arabica*	Catuai IAC144	India	Karnataka State	2019	861	13.3739	75.57583	14
U-1540/PURN11725	*Coffea arabica*	---	Puerto Rico	Utuado	2014	256	18.266	-66.70366	15
U-1541/PURN11726	*Coffea arabica*	Bourbon	Puerto Rico	Utuado	2014	256	18.266	-66.70366	10
WCRH1	*Coffea arabica*	Centroamericano H1	Honduras	Santa Barbara	2019	962	14.9989	-88.055	25
WCRH2	*Coffea arabica*	Oro Azteca	Honduras	Santa Barbara	2019	962	14.9989	-88.055	10
BA 1-10	*Coffea arabica*	Typica	Jamaica	St. Ann	2016	566	18.2277	-77.38432	10
BA 1-11	*Coffea arabica*	Typica	Jamaica	St. Ann	2016	566	18.2277	-77.38432	10
BA 1-12	*Coffea arabica*	Typica	Jamaica	St. Ann	2016	566	18.2277	-77.38432	10
BA 1-3	*Coffea arabica*	Typica	Jamaica	St. Ann	2016	566	18.2277	-77.38432	10
BA 1-4	*Coffea arabica*	Typica	Jamaica	St. Ann	2016	566	18.2277	-77.38432	10
BA 1-5	*Coffea arabica*	Typica	Jamaica	St. Ann	2016	566	18.2277	-77.38432	10
BA 1-6	*Coffea arabica*	Typica	Jamaica	St. Ann	2016	566	18.2277	-77.38432	10
BA 1-7	*Coffea arabica*	Typica	Jamaica	St. Ann	2016	566	18.2277	-77.38432	10
BA 1-8	*Coffea arabica*	Typica	Jamaica	St. Ann	2016	566	18.2277	-77.38432	10
BA 1-9	*Coffea arabica*	Typica	Jamaica	St. Ann	2016	566	18.2277	-77.38432	10
BB-4	*Coffea arabica*	Typica	Jamaica	Portland	2016	908	18.1088	-76.7006	10

BB-6	*Coffea arabica*	Typica	Jamaica	Portland	2016	894	18.1098	-76.69992	10
BB-7	*Coffea arabica*	Typica	Jamaica	Portland	2016	894	18.1102	-76.69988	10
BB 1-12	*Coffea arabica*	Typica	Jamaica	Portland	2016	897	18.1095	-76.70008	10
BB 1-9	*Coffea arabica*	Typica	Jamaica	Portland	2016	897	18.1095	-76.70008	10
BC1-11	*Coffea arabica*	Typica	Jamaica	Clarendon	2016	551	18.211	-77.37487	22
BC1-12	*Coffea arabica*	Typica	Jamaica	Clarendon	2016	551	18.211	-77.37487	23
BC1-2	*Coffea arabica*	Typica	Jamaica	Clarendon	2016	551	18.211	-77.37487	27
BC1-3	*Coffea arabica*	Typica	Jamaica	Clarendon	2016	551	18.211	-77.37487	37
BC1-4	*Coffea arabica*	Typica	Jamaica	Clarendon	2016	551	18.211	-77.37487	37
BC1-6	*Coffea arabica*	Typica	Jamaica	Clarendon	2016	551	18.211	-77.37487	37
BC1-7	*Coffea arabica*	Typica	Jamaica	Clarendon	2016	551	18.211	-77.37487	10
BC1-8	*Coffea arabica*	Typica	Jamaica	Clarendon	2016	551	18.211	-77.37487	22
BC10	*Coffea arabica*	Typica	Jamaica	Clarendon	2016	566	18.2194	-77.38527	22
BC11	*Coffea arabica*	Typica	Jamaica	Clarendon	2016	566	18.2194	-77.38533	22
BC12	*Coffea arabica*	Typica	Jamaica	Clarendon	2016	564	18.2194	-77.38533	22
BC3	*Coffea arabica*	Typica	Jamaica	Clarendon	2016	551	18.2098	-77.37442	22
BC4	*Coffea arabica*	Typica	Jamaica	Clarendon	2016	552	18.209	-77.37455	22
BC5	*Coffea arabica*	Typica	Jamaica	Clarendon	2016	551	18.2115	-77.36625	22
BC6	*Coffea arabica*	Typica	Jamaica	Clarendon	2016	548	18.2115	-77.36607	22
BC7	*Coffea arabica*	Typica	Jamaica	Clarendon	2016	549	18.2119	-77.36615	22
BC8	*Coffea arabica*	Typica	Jamaica	Clarendon	2016	554	18.2123	-77.36603	22
CE-11	*Coffea canephora*	Robusta	Jamaica	St. Andrew	2016	773	18.0529	-76.72133	10
CE-2	*Coffea arabica*	Typica	Jamaica	St. Andrew	2016	819	18.054	-76.7202	10
CE-4	*Coffea arabica*	Typica	Jamaica	St. Andrew	2016	811	18.0538	-76.71995	10
CE-5	*Coffea arabica*	---	Jamaica	St. Andrew	2016	807	18.0537	-76.7194	10
CE-6	*Coffea arabica*	---	Jamaica	St. Andrew	2016	807	18.0537	-76.7194	10
CE-9	*Coffea arabica*	Typica	Jamaica	St. Andrew	2016	787	18.0529	-76.72065	10
CE1-1	*Coffea arabica*	Typica	Jamaica	St. Andrew	2016	817	18.0541	-76.7203	10

CE1-11	*Coffea arabica*	Typica	Jamaica	St. Andrew	2016	817	18.0541	-76.7203	10
CE1-13	*Coffea arabica*	Typica	Jamaica	St. Andrew	2016	817	18.0541	-76.7203	10
CE1-14	*Coffea arabica*	Typica	Jamaica	St. Andrew	2016	817	18.0541	-76.7203	10
CE1-15	*Coffea arabica*	Typica	Jamaica	St. Andrew	2016	817	18.0541	-76.7203	10
CE1-3	*Coffea arabica*	Typica	Jamaica	St. Andrew	2016	817	18.0541	-76.7203	10
CE1-4	*Coffea arabica*	Typica	Jamaica	St. Andrew	2016	817	18.0541	-76.7203	10
CE1-5	*Coffea arabica*	Typica	Jamaica	St. Andrew	2016	817	18.0541	-76.7203	10
CE1-6	*Coffea arabica*	Typica	Jamaica	St. Andrew	2016	817	18.0541	-76.7203	10
CE1-7	*Coffea arabica*	Typica	Jamaica	St. Andrew	2016	817	18.0541	-76.7203	10
CE1-8	*Coffea arabica*	Typica	Jamaica	St. Andrew	2016	817	18.0541	-76.7203	10
CE1-9	*Coffea arabica*	Typica	Jamaica	St. Andrew	2016	817	18.0541	-76.7203	10
DW-10	*Coffea arabica*	Catimor	Jamaica	St. Thomas	2016	472	17.9847	-76.57055	10
DW-12	*Coffea arabica*	Typica	Jamaica	St. Thomas	2016	470	17.9848	-76.57067	10
DW-4	*Coffea arabica*	Catimor	Jamaica	St. Thomas	2016	473	17.985	-76.57078	10
DW-5	*Coffea arabica*	Catimor	Jamaica	St. Thomas	2016	481	17.9849	-76.57088	10
DW-7	*Coffea arabica*	Catimor	Jamaica	St. Thomas	2016	482	17.9846	-76.57075	10
DW-9	*Coffea arabica*	Catimor	Jamaica	St. Thomas	2016	472	17.9847	-76.57055	10
DW1-11	*Coffea arabica*	Catimor	Jamaica	St. Thomas	2016	468	17.9849	-76.57023	10
DW1-3	*Coffea arabica*	Catimor	Jamaica	St. Thomas	2016	468	17.9849	-76.57023	10
DW1-5	*Coffea arabica*	Catimor	Jamaica	St. Thomas	2016	468	17.9849	-76.57023	10
DW1-7	*Coffea arabica*	Catimor	Jamaica	St. Thomas	2016	468	17.9849	-76.57023	34
DW1-9	*Coffea arabica*	Catimor	Jamaica	St. Thomas	2016	468	17.9849	-76.57023	10
EL-10	*Coffea arabica*	Catimor	Jamaica	St. Elizabeth	2016	453	18.2224	-77.75372	10
EL-11	*Coffea arabica*	Typica	Jamaica	St. Elizabeth	2016	453	18.2224	-77.75372	10
EL-3	*Coffea arabica*	---	Jamaica	St. Elizabeth	2016	450	18.2224	-77.75385	22
EL-5	*Coffea arabica*	---	Jamaica	St. Elizabeth	2016	450	18.2225	-77.7538	10
EL-6	*Coffea arabica*	Typica	Jamaica	St. Elizabeth	2016	449	18.2224	-77.75375	10
EL-8	*Coffea arabica*	---	Jamaica	St. Elizabeth	2016	451	18.2224	-77.75372	22

EL-9	*Coffea arabica*	---	Jamaica	St. Elizabeth	2016	452	18.2224	-77.75373	22
EL1-5	*Coffea arabica*	Typica	Jamaica	St. Elizabeth	2016	448	18.2224	-77.75387	26
EL1-7	*Coffea arabica*	Typica	Jamaica	St. Elizabeth	2016	448	18.2224	-77.75387	26
EL1-8	*Coffea arabica*	Typica	Jamaica	St. Elizabeth	2016	448	18.2224	-77.75387	10
EL1-9	*Coffea arabica*	Typica	Jamaica	St. Elizabeth	2016	448	18.2224	-77.75387	10
GW-10	*Coffea arabica*	Typica	Jamaica	St. Andrew	2016	1088	18.0719	-76.7263	10
GW-12	*Coffea arabica*	Typica	Jamaica	St. Andrew	2016	1083	18.0718	-76.726	22
GW-3	*Coffea arabica*	Typica	Jamaica	St. Andrew	2016	1076	18.0713	-76.72538	22
GW-4	*Coffea arabica*	Typica	Jamaica	St. Andrew	2016	1074	18.0708	-76.72563	10
GW-5	*Coffea arabica*	---	Jamaica	St. Andrew	2016	1083	18.0718	-76.72575	22
GW-7	*Coffea arabica*	Typica	Jamaica	St. Andrew	2016	1093	18.0728	-76.7255	22
GW-8	*Coffea arabica*	Typica	Jamaica	St. Andrew	2016	1091	18.0726	-76.72572	22
GW-9	*Coffea arabica*	---	Jamaica	St. Andrew	2016	1093	18.0723	-76.72613	22
GW1-2	*Coffea arabica*	Typica	Jamaica	St. Andrew	2016	1080	18.0715	-76.72547	10
GW1-3	*Coffea arabica*	Typica	Jamaica	St. Andrew	2016	1080	18.0715	-76.72547	10
GW1-5	*Coffea arabica*	Typica	Jamaica	St. Andrew	2016	1080	18.0715	-76.72547	10
GW1-6	*Coffea arabica*	Typica	Jamaica	St. Andrew	2016	1080	18.0715	-76.72547	10
GW1-9	*Coffea arabica*	Typica	Jamaica	St. Andrew	2016	1080	18.0715	-76.72547	10
HV-10	*Coffea arabica*	Typica	Jamaica	Hanover	2016	143	18.3261	-77.92805	2
HV-3	*Coffea arabica*	Typica	Jamaica	Hanover	2016	150	18.3261	-77.92802	2
HV-4	*Coffea arabica*	Typica	Jamaica	Hanover	2016	147	18.3262	-77.9279	30
HV-5	*Coffea arabica*	Typica	Jamaica	Hanover	2016	145	18.326	-77.92788	10
HV-7	*Coffea arabica*	Typica	Jamaica	Hanover	2016	144	18.3258	-77.92792	10
HV-8	*Coffea arabica*	Typica	Jamaica	Hanover	2016	149	18.3259	-77.9281	10
HV-9	*Coffea arabica*	Typica	Jamaica	Hanover	2016	146	18.326	-77.9281	42
HV1-10	*Coffea arabica*	Typica	Jamaica	Hanover	2016	150	18.3263	-77.928	10
HV1-12	*Coffea arabica*	Typica	Jamaica	Hanover	2016	150	18.3263	-77.928	42
HV1-2	*Coffea arabica*	Typica	Jamaica	Hanover	2016	150	18.3263	-77.928	10

HV1-7	*Coffea arabica*	Typica	Jamaica	Hanover	2016	150	18.3263	-77.928	10
HV1-8	*Coffea arabica*	Typica	Jamaica	Hanover	2016	150	18.3263	-77.928	10
KP-10	*Coffea arabica*	Typica	Jamaica	Westmoreland	2016	368	18.2579	-77.94797	22
KP-11	*Coffea arabica*	Typica	Jamaica	Westmoreland	2016	362	18.2579	-77.94768	10
KP-12	*Coffea arabica*	Typica	Jamaica	Westmoreland	2016	366	18.2579	-77.94742	22
KP-2	*Coffea arabica*	---	Jamaica	Westmoreland	2016	380	18.2556	-77.9424	22
KP-3	*Coffea arabica*	---	Jamaica	Westmoreland	2016	373	18.2556	-77.94225	22
KP-4	*Coffea arabica*	Typica	Jamaica	Westmoreland	2016	377	18.2555	-77.94252	22
KP-6	*Coffea arabica*	Catimor	Jamaica	Westmoreland	2016	381	18.2552	-77.9425	21
KP-7	*Coffea arabica*	Catimor	Jamaica	Westmoreland	2016	376	18.2585	-77.94852	10
KP-8	*Coffea arabica*	Typica	Jamaica	Westmoreland	2016	373	18.2583	-77.94823	10
KP1-1	*Coffea arabica*	Typica	Jamaica	Westmoreland	2016	347	18.2617	-77.94552	10
KP1-10	*Coffea arabica*	Typica	Jamaica	Westmoreland	2016	347	18.2617	-77.94552	24
KP1-11	*Coffea arabica*	Typica	Jamaica	Westmoreland	2016	347	18.2617	-77.94552	10
KP1-12	*Coffea arabica*	Typica	Jamaica	Westmoreland	2016	347	18.2617	-77.94552	22
KP1-2	*Coffea arabica*	Typica	Jamaica	Westmoreland	2016	347	18.2617	-77.94552	10
KP1-3	*Coffea arabica*	Typica	Jamaica	Westmoreland	2016	347	18.2617	-77.94552	10
KP1-4	*Coffea arabica*	Typica	Jamaica	Westmoreland	2016	347	18.2617	-77.94552	10
KP1-5	*Coffea arabica*	Typica	Jamaica	Westmoreland	2016	347	18.2617	-77.94552	37
KP1-6	*Coffea arabica*	Typica	Jamaica	Westmoreland	2016	347	18.2617	-77.94552	13
KP1-7	*Coffea arabica*	Typica	Jamaica	Westmoreland	2016	347	18.2617	-77.94552	22
KP1-8	*Coffea arabica*	Typica	Jamaica	Westmoreland	2016	347	18.2617	-77.94552	10
KP1-9	*Coffea arabica*	Typica	Jamaica	Westmoreland	2016	347	18.2617	-77.94552	10
MB-10	*Coffea arabica*	Typica	Jamaica	St. Thomas	2016	355	17.9748	-76.55457	10
MB-11	*Coffea arabica*	Typica	Jamaica	St. Thomas	2016	356	17.9748	-76.55432	10
MB-2	*Coffea arabica*	Typica	Jamaica	St. Thomas	2016	368	17.9752	-76.5543	10
MB-3	*Coffea arabica*	Typica	Jamaica	St. Thomas	2016	364	17.9752	-76.5543	10
MB-5	*Coffea arabica*	Typica	Jamaica	St. Thomas	2016	362	17.975	-76.5544	10

MB-6	*Coffea arabica*	Typica	Jamaica	St. Thomas	2016	359	17.9749	-76.55432	10
MB-7	*Coffea arabica*	Typica	Jamaica	St. Thomas	2016	359	17.9749	-76.55432	10
MB-8	*Coffea arabica*	Typica	Jamaica	St. Thomas	2016	359	17.975	-76.5545	10
MB-9	*Coffea arabica*	Typica	Jamaica	St. Thomas	2016	359	17.975	-76.5545	10
MB1-10	*Coffea arabica*	Typica	Jamaica	St. Thomas	2016	374	17.9754	-76.55448	10
MB1-12	*Coffea arabica*	Typica	Jamaica	St. Thomas	2016	374	17.9754	-76.55448	10
MB1-2	*Coffea arabica*	Typica	Jamaica	St. Thomas	2016	374	17.9754	-76.55448	10
MB1-4	*Coffea arabica*	Typica	Jamaica	St. Thomas	2016	374	17.9754	-76.55448	10
MB1-5	*Coffea arabica*	Typica	Jamaica	St. Thomas	2016	374	17.9754	-76.55448	10
MB1-8	*Coffea arabica*	Typica	Jamaica	St. Thomas	2016	374	17.9754	-76.55448	10
MB1-9	*Coffea arabica*	Typica	Jamaica	St. Thomas	2016	374	17.9754	-76.55448	10
MF-10	*Coffea arabica*	Typica	Jamaica	St. Andrew	2016	452	18.109	-76.80393	10
MF-11	*Coffea arabica*	Typica	Jamaica	St. Andrew	2016	447	18.1093	-76.80387	10
MF-2	*Coffea arabica*	Typica	Jamaica	St. Andrew	2016	453	18.1091	-76.80405	10
MF-4	*Coffea arabica*	Typica	Jamaica	St. Andrew	2016	464	18.109	-76.80447	10
MF-5	*Coffea arabica*	Typica	Jamaica	St. Andrew	2016	466	18.1089	-76.8044	10
MF-7	*Coffea arabica*	Typica	Jamaica	St. Andrew	2016	467	18.1088	-76.80437	10
MF-8	*Coffea arabica*	Typica	Jamaica	St. Andrew	2016	455	18.1089	-76.80388	10
MF-9	*Coffea arabica*	Typica	Jamaica	St. Andrew	2016	453	18.1089	-76.8038	10
MF1-10	*Coffea arabica*	Typica	Jamaica	St. Andrew	2016	449	18.1092	-76.80407	22
MF1-11	*Coffea arabica*	Typica	Jamaica	St. Andrew	2016	449	18.1092	-76.80407	20
MF1-5	*Coffea arabica*	Typica	Jamaica	St. Andrew	2016	449	18.1092	-76.80407	10
MF1-6	*Coffea arabica*	Typica	Jamaica	St. Andrew	2016	449	18.1092	-76.80407	10
MF1-7	*Coffea arabica*	Typica	Jamaica	St. Andrew	2016	449	18.1092	-76.80407	22
MH1-11	*Coffea arabica*	Catimor	Jamaica	St. Thomas	2016	825	18.01	-76.59517	10
MH1-2	*Coffea arabica*	Catimor	Jamaica	St. Thomas	2016	825	18.01	-76.59517	10
MH1-4	*Coffea arabica*	Catimor	Jamaica	St. Thomas	2016	825	18.01	-76.59517	10
MH1-6	*Coffea arabica*	Catimor	Jamaica	St. Thomas	2016	825	18.01	-76.59517	10

MH1-7	*Coffea arabica*	Catimor	Jamaica	St. Thomas	2016	825	18.01	-76.59517	10
MH1-8	*Coffea arabica*	Catimor	Jamaica	St. Thomas	2016	825	18.01	-76.59517	10
MH1-9	*Coffea arabica*	Catimor	Jamaica	St. Thomas	2016	825	18.01	-76.59517	10
PH-10	*Coffea arabica*	Typica	Jamaica	St. Mary	2016	240	18.2865	-76.9878	10
PH-11	*Coffea arabica*	Typica	Jamaica	St. Mary	2016	234	18.2865	-76.98812	10
PH-12	*Coffea arabica*	---	Jamaica	St. Mary	2016	240	18.2865	-76.98785	10
PH-2	*Coffea arabica*	Typica	Jamaica	St. Mary	2016	334	18.2842	-76.9849	10
PH-3	*Coffea arabica*	Typica	Jamaica	St. Mary	2016	335	18.2842	-76.98485	38
PH-4	*Coffea arabica*	Typica	Jamaica	St. Mary	2016	345	18.2838	-76.98488	10
PH-6	*Coffea arabica*	Typica	Jamaica	St. Mary	2016	344	18.2838	-76.98468	10
PH-9	*Coffea arabica*	Typica	Jamaica	St. Mary	2016	240	18.2864	-76.98785	10
PH1-11	*Coffea arabica*	Typica	Jamaica	St. Mary	2016	339	18.2843	-76.98495	38
PH1-12	*Coffea arabica*	Typica	Jamaica	St. Mary	2016	339	18.2843	-76.98495	10
PH1-2	*Coffea arabica*	Typica	Jamaica	St. Mary	2016	339	18.2843	-76.98495	38
PH1-5	*Coffea arabica*	Typica	Jamaica	St. Mary	2016	339	18.2843	-76.98495	38
PH1-6	*Coffea arabica*	Typica	Jamaica	St. Mary	2016	339	18.2843	-76.98495	38
PH1-7	*Coffea arabica*	Typica	Jamaica	St. Mary	2016	339	18.2843	-76.98495	38
PH1-8	*Coffea arabica*	Typica	Jamaica	St. Mary	2016	339	18.2843	-76.98495	38
PH1-9	*Coffea arabica*	Typica	Jamaica	St. Mary	2016	339	18.2843	-76.98495	38
SJ1-1	*Coffea arabica*	Typica	Jamaica	St. James	2016	159	18.3347	-76.91097	10
SJ1-6	*Coffea arabica*	Typica	Jamaica	St. James	2016	159	18.3347	-76.91097	10
TP-11	*Coffea arabica*	---	Jamaica	Trelawny	2016	176	18.3794	-77.63283	10
TP-3	*Coffea arabica*	---	Jamaica	Trelawny	2016	165	18.3792	-77.63308	10
TP-4	*Coffea arabica*	---	Jamaica	Trelawny	2016	171	18.3792	-77.63281	10
TP-8	*Coffea arabica*	---	Jamaica	Trelawny	2016	177	18.3794	-77.63275	10
TP-9	*Coffea arabica*	---	Jamaica	Trelawny	2016	166	18.3796	-77.63278	10
TP1-1	*Coffea arabica*	---	Jamaica	Trelawny	2016	190	18.3795	-77.63317	10
TP1-12	*Coffea arabica*	---	Jamaica	Trelawny	2016	190	18.3795	-77.63317	10

TQ-10	*Coffea arabica*	Typica	Jamaica	Portland	2016	126	18.1861	-76.68143	10
TQ-11	*Coffea arabica*	Typica	Jamaica	Portland	2016	125	18.1862	-76.68155	10
TQ-12	*Coffea arabica*	Typica	Jamaica	Portland	2016	122	18.1864	-76.68125	28
TQ-2	*Coffea arabica*	Typica	Jamaica	Portland	2016	124	18.1856	-76.68175	10
TQ-3	*Coffea arabica*	Typica	Jamaica	Portland	2016	124	18.1856	-76.68175	10
TQ-4	*Coffea arabica*	Typica	Jamaica	Portland	2016	123	18.1857	-76.68163	10
TQ-5	*Coffea arabica*	Typica	Jamaica	Portland	2016	128	18.1856	-76.68187	10
TQ-6	*Coffea arabica*	Typica	Jamaica	Portland	2016	126	18.1857	-76.6818	10
TQ-7	*Coffea arabica*	Typica	Jamaica	Portland	2016	126	18.1858	-76.68167	10
TQ-8	*Coffea arabica*	Typica	Jamaica	Portland	2016	125	18.1858	-76.68163	10
TQ1-4	*Coffea arabica*	Typica	Jamaica	Portland	2016	124	18.1856	-76.68175	10
TQ1-6	*Coffea arabica*	Typica	Jamaica	Portland	2016	124	18.1856	-76.68175	10
TQ1-7	*Coffea arabica*	Typica	Jamaica	Portland	2016	124	18.1856	-76.68175	10
TQ1-9	*Coffea arabica*	Typica	Jamaica	Portland	2016	124	18.1856	-76.68175	10
WF-10	*Coffea arabica*	Typica	Jamaica	Portland	2016	---	18.1202	-76.71438	22
WF-11	*Coffea arabica*	Typica	Jamaica	Portland	2016	---	18.1199	-76.71428	22
WF-2	*Coffea arabica*	Catimor	Jamaica	Portland	2016	---	18.1192	-76.71513	22
WF-3	*Coffea arabica*	Catimor	Jamaica	Portland	2016	---	18.1194	-76.715	22
WF-4	*Coffea arabica*	Catimor	Jamaica	Portland	2016	---	18.1197	-76.71498	22
WF-5	*Coffea arabica*	Typica	Jamaica	Portland	2016	---	18.12	-76.71505	10
WF-7	*Coffea arabica*	Typica	Jamaica	Portland	2016	---	18.1202	-76.7147	10
WF-9	*Coffea arabica*	Catimor	Jamaica	Portland	2016	---	18.1201	-76.71442	24
WF1-10	*Coffea arabica*	Catimor	Jamaica	Portland	2016	414	18.1192	-76.71513	10
WF1-11	*Coffea arabica*	Catimor	Jamaica	Portland	2016	414	18.1192	-76.71513	22
WF1-12	*Coffea arabica*	Catimor	Jamaica	Portland	2016	414	18.1192	-76.71513	22
WF1-4	*Coffea arabica*	Catimor	Jamaica	Portland	2016	414	18.1192	-76.71513	10
WF1-6	*Coffea arabica*	Catimor	Jamaica	Portland	2016	414	18.1192	-76.71513	10
WF1-7	*Coffea arabica*	Catimor	Jamaica	Portland	2016	414	18.1192	-76.71513	10

WF1-8	*Coffea arabica*	Catimor	Jamaica	Portland	2016	414	18.1192	-76.71513	10
WF1-9	*Coffea arabica*	Catimor	Jamaica	Portland	2016	414	18.1192	-76.71513	10
BA 2 (A) [R]	*Coffea arabica*	Typica	Jamaica	St. Ann	2018	556	18.2276	-77.3842	10
BB-6 [R]	*Coffea arabica*	Typica	Jamaica	Portland	2018	894	18.1098	-76.69992	22
BB-7 (A) [R]	*Coffea arabica*	Typica	Jamaica	Portland	2018	894	18.1102	-76.69988	10
DW-3 (B) [R]	*Coffea arabica*	Catimor	Jamaica	St. Thomas	2018	472	17.9851	-76.57073	10
DW-8 (A) [R]	*Coffea arabica*	Catimor	Jamaica	St. Thomas	2018	478	17.9845	-76.57052	10
EL-10 [R]	*Coffea arabica*	Catimor	Jamaica	St. Elizabeth	2018	453	18.2224	-77.75372	27
EL-11 (A) [R]	*Coffea arabica*	Typica	Jamaica	St. Elizabeth	2018	453	18.2224	-77.75372	27
HV-5 [R]	*Coffea arabica*	Typica	Jamaica	Hanover	2018	145	18.326	-77.92788	10
MF-6 [R]	*Coffea arabica*	Typica	Jamaica	St. Andrew	2018	466	18.1089	-76.80457	10
MH-12 [R]	*Coffea arabica*	Catimor	Jamaica	St. Thomas	2018	835	18.0104	-76.59578	10
TP-6 (B) [R]	*Coffea arabica*	---	Jamaica	Trelawny	2018	149	18.3792	-77.63286	10
TQ-1 [R]	*Coffea arabica*	Typica	Jamaica	Portland	2018	124	18.1856	-76.68175	10
TQ-3 [R]	*Coffea arabica*	Typica	Jamaica	Portland	2018	124	18.1856	-76.68175	10
TQ-8 [R]	*Coffea arabica*	Typica	Jamaica	Portland	2018	125	18.1858	-76.68163	10
U1771 a	*Coffea arabica*	Feral	USA	Maui	2020	329	20.9992	-156.5656	36
U1771 b	*Coffea arabica*	Feral	USA	Maui	2020	329	20.9992	-156.5656	36
U1772 a	*Coffea arabica*	Feral	USA	Maui	2020	288	20.9142	-156.3306	10
U1772 b	*Coffea arabica*	Feral	USA	Maui	2020	288	20.9142	-156.3306	36
U1773 a	*Coffea arabica*	Typica	USA	Hawaii Island	2020	140	19.5678	-155.9356	10
U1773 b	*Coffea arabica*	Typica	USA	Hawaii Island	2020	140	19.5678	-155.9356	10
U1774 A	*Coffea arabica*	Feral	USA	Hawaii Island	2020	140	19.5583	-155.9293	36
U1774 B	*Coffea arabica*	Typica	USA	Hawaii Island	2020	400	19.56	-155.9241	36
U1774 C	*Coffea arabica*	Typica	USA	Hawaii Island	2020	140	19.6419	-155.9969	36
U1774 D	*Coffea arabica*	Typica	USA	Hawaii Island	2020	140	19.6465	-155.9491	36
PUR018142	*Coffea arabica*	---	Brazil	Sao Paulo	1976	779	-23.836	-46.69251	35
PUR018143	*Coffea arabica*	---	Brazil	Sao Paulo	1976	575	-20.906	-48.63872	2

PUR018146	*Coffea arabica*	---	Brazil	Sao Paulo	1975	499	-22	-49	2
PUR018147	*Coffea arabica*	---	Brazil	Sao Paulo	1975	683	-22.906	-47.06083	11
PUR018150	*Coffea arabica*	---	Brazil	Sao Paulo	1976	522	-21.009	-48.22167	2
PUR018151	*Coffea arabica*	---	Brazil	Sao Paulo	1976	522	-21.009	-48.22167	2
PUR018152	*Coffea arabica*	---	Brazil	Minas Gerais	1976	244	-21.531	-42.64035	35
PUR018155	*Coffea arabica*	---	Brazil	Sao Paulo	1976	852	-22.952	-46.54186	41
PUR018158	*Coffea arabica*	---	Brazil	Sao Paulo	1976	843	-21.528	-46.64366	2
PUR018163	*Coffea arabica*	---	Brazil	Mato Grosso	1988	803	-15.464	-55.74986	2
PUR018165	*Coffea arabica*	---	Brazil	Sao Paulo	1983	731	-22.732	-48.57232	2
PUR018167	*Coffea arabica*	---	Brazil	Sao Paulo	1983	763	-22.254	-47.82125	2
PUR018168	*Coffea arabica*	---	Brazil	Sao Paulo	1995	499	-22	-49	35
PUR018170	*Coffea arabica*	---	Brazil	Sao Paulo	1995	538	-22.725	-47.64917	35
PUR018171	*Coffea arabica*	---	Brazil	Minas Gerais	1986	679	-19.787	-45.68467	35
PUR018172	*Coffea arabica*	---	Brazil	Minas Gerais	1983	909	-21.243	-44.9992	2
PUR018175	*Coffea arabica*	---	Brazil	---	1981	---	-9.6757	-54.46973	2
PUR018178	*Coffea arabica*	---	Brazil	Minas Gerais	1986	909	-21.243	-44.9992	40
PUR018179	*Coffea arabica*	---	Myanmar	Burma	1971	1083	22.0283	96.47066	2
PUR018244	*Coffea liberica*	---	Indonesia	West Java	1952	261	-6.5963	106.7972	38
U1618	*Coffea arabica*	---	Indonesia	Sumatra	2016	1700	4.5823	96.88971	1
PUR018237	*Coffea arabica*	---	Nigeria	Abia State	1984	128	5.52627	7.489589	16

Abbreviations are: SNNPR, Southern Nations, Nationalities, and Peoples' Region; DRC, Democratic Republic of the Congo.

Table S2. Three newly generated SSRs used in this study.

Primer Code	Forward (5'-3') [a]	Reverse (5'-3')	Repeat motif	Product size
SSR110	AAAGTCTATTAGTGTGAGGTGGTT	GTGGCCACTGAGCAATTTGC	AG (14)	190
SSR161	TGACTATGAAACCTCGTCGTCA	AGTGGATAAAGGAAGAGGAATCGG	AAG (21)	208
SSR183	TTCCTGCTTCCATCGTTCCT	AACTACACACGTTGTATGTATGCA	AC (21)	182

[a] Each 5’3’ forward primer is labeled with the universal M13 fluorescently primer: TGTAAAACGACGGCCAGT.

Table S3. Input file of *Hemileia vastatrix* data

IND[a]	Pop[b]	110^{1}	110^{2}	126^{1}	126^{2}	148^{1}	148^{2}	150^{1}	150^{2}	154^{1}	154^{2}	159^{1}	159^{2}	161^{1}	161^{2}	164^{1}	164^{2}	166^{1}	166^{2}	180^{1}	180^{2}	183^{1}	183^{2}
A	ETH	209	209	188	196	179	188	142	154	165	165	160	175	225	234	187	193	210	216	207	207	198	203
AB	ETH	209	209	188	196	179	188	142	154	165	165	160	175	225	234	187	193	210	216	207	207	198	203
AD	ETH	209	209	188	196	179	188	142	154	165	165	160	175	225	234	187	193	210	216	207	207	198	203
AE	ETH	209	209	188	196	179	188	142	154	165	165	160	175	225	234	187	193	210	216	207	207	198	203
AG	ETH	209	209	188	196	179	188	142	154	165	165	160	175	206	225	187	193	210	216	207	207	198	203
AH	ETH	209	209	188	196	179	188	142	154	165	165	160	175	225	234	187	193	210	216	207	207	198	203
AJ	ETH	209	209	188	196	179	188	142	154	165	165	160	175	225	234	187	193	210	216	207	207	198	203
AK	ETH	209	209	188	196	179	188	142	154	165	165	160	175	225	234	187	193	210	216	207	207	198	203
AL	ETH	209	209	188	196	179	188	142	154	165	165	160	175	225	234	187	193	210	216	207	207	198	203
AM	ETH	209	209	188	196	179	188	142	154	165	165	160	175	225	234	187	193	210	216	207	207	198	203
AO	ETH	209	209	188	196	179	188	142	154	165	165	160	175	225	234	187	193	210	216	207	207	198	203
AR	ETH	209	209	188	196	179	188	142	154	165	165	160	175	225	234	187	193	210	216	207	207	198	203
AS	ETH	209	209	188	196	179	188	142	154	165	165	160	175	225	234	187	193	210	216	207	207	198	203
AT	ETH	209	209	188	196	179	188	142	154	165	165	160	175	225	234	187	193	210	216	207	207	198	203
AV	ETH	209	209	188	196	179	188	142	154	165	165	160	175	225	234	187	193	210	216	207	207	198	203
AW	ETH	209	209	188	196	179	188	142	154	165	165	160	175	206	225	187	193	210	216	207	207	198	203
AY	ETH	209	209	188	196	179	188	142	154	165	165	160	175	225	234	187	193	210	216	207	207	198	203
AZ	ETH	209	209	188	196	179	188	142	154	165	165	160	175	206	225	187	193	210	216	207	207	198	203
BA	ETH	209	209	188	196	179	188	142	154	165	165	160	175	225	234	187	193	210	216	207	207	198	203
BB	ETH	209	209	188	196	179	188	142	154	165	165	160	175	225	234	187	193	210	216	207	207	198	203
BC	ETH	209	209	188	196	179	188	142	154	165	165	160	175	225	234	187	193	210	216	207	207	198	203
BD	ETH	209	209	188	196	179	188	142	154	165	165	160	175	225	234	187	193	210	216	207	207	198	203
BE	ETH	209	209	188	196	179	188	142	154	165	165	160	175	225	234	187	193	210	216	207	207	198	203
BI	ETH	209	209	188	196	179	188	142	154	165	165	160	175	225	234	187	193	210	216	207	207	198	203
C	ETH	209	209	188	196	179	188	142	154	165	165	160	175	225	234	187	193	210	216	207	207	198	203
CN	ETH	209	209	188	196	179	188	142	154	165	165	160	175	225	234	187	193	210	216	207	207	198	203

CO	ETH	209	209	188	196	179	188	142	154	165	165	160	175	225	234	187	193	210	216	207	207	198	203
CQ	ETH	209	209	188	196	179	188	142	154	165	165	160	175	206	225	187	193	210	216	207	207	198	203
CS	ETH	209	209	188	196	179	188	142	154	165	165	160	175	225	234	187	193	210	216	207	207	198	203
CU	ETH	209	209	188	196	179	188	142	154	165	165	160	175	225	234	187	193	210	216	207	207	198	203
D	ETH	209	209	188	196	179	188	142	154	165	165	160	175	225	234	187	193	210	216	207	207	198	203
N	ETH	209	209	188	196	179	188	142	154	165	165	160	175	225	234	187	193	210	216	207	207	198	203
O	ETH	209	209	188	196	179	188	142	154	165	165	160	175	225	234	187	193	210	216	207	207	198	203
Q	ETH	209	209	188	196	179	188	142	154	165	165	160	175	225	234	187	193	210	216	207	207	198	203
T	ETH	209	209	188	196	179	188	142	154	165	165	160	175	225	234	187	193	210	216	207	207	198	203
U	ETH	209	209	188	196	179	188	142	154	165	165	160	175	225	234	187	193	210	216	207	207	198	203
MCA8388	ETH	209	209	188	196	179	188	142	154	165	165	160	175	225	234	187	193	210	216	207	207	198	203
MCA8388	ETH	209	209	188	196	179	188	142	154	165	165	160	175	225	234	187	193	210	216	207	207	198	203
MCA8388	ETH	209	209	188	196	179	188	142	154	165	165	160	175	225	234	187	193	210	216	207	207	198	203
MCA8388	ETH	209	209	188	196	179	188	142	154	165	165	160	175	225	234	187	193	210	216	207	207	198	203
MCA8388	ETH	209	209	188	196	179	188	142	154	165	165	160	175	225	234	187	193	210	216	207	207	198	203
MCA8392	ETH	209	209	188	196	179	188	142	154	165	165	160	175	225	234	187	193	210	216	207	207	198	203
MCA8392	ETH	209	209	188	196	179	188	142	154	165	165	160	175	225	234	187	193	210	216	207	207	198	203
MCA8392	ETH	209	209	188	196	179	188	142	154	165	165	160	175	225	234	187	193	210	216	207	207	198	203
MCA8392	ETH	209	209	188	196	179	188	142	154	165	165	160	175	225	234	187	193	210	216	207	207	198	203
MCA8392	ETH	209	209	188	196	179	188	142	154	165	165	160	175	225	234	187	193	210	216	207	207	198	203
PUR054576	CMRN	185	199	186	194	179	194	139	154	156	156	160	175	209	237	184	187	204	210	188	188	186	207
RAK421	CMRN	185	199	186	194	179	194	139	154	156	156	160	175	209	237	184	187	204	210	188	188	186	207
PUR054577	CMRN	185	199	186	194	179	194	139	154	156	156	160	175	209	237	184	187	204	210	188	188	186	207
PUR054578	CMRN	185	199	186	194	179	194	139	154	156	156	160	175	209	237	184	187	204	210	188	188	186	207
PUR045847	CMRN	185	199	186	194	179	194	139	154	156	156	160	175	209	237	184	187	204	210	188	188	186	207
PHV001	PAN	209	209	188	194	179	188	142	154	162	165	160	175	225	234	187	193	210	216	207	207	198	203
PHV006	PAN	209	209	188	194	179	188	142	154	162	165	160	175	225	234	187	193	210	216	207	207	198	203
PHV008	PAN	209	209	188	194	179	188	142	154	162	165	160	175	225	234	187	193	210	216	207	207	198	203

PHV009	PAN	209	209	188	194	179	188	142	154	162	165	160	175	225	234	187	193	210	216	207	207	198	203
PHV011	PAN	209	209	188	194	179	188	142	154	162	165	160	175	225	234	187	193	210	216	207	207	198	203
PHV013	PAN	209	209	188	194	179	188	142	154	162	165	160	175	225	234	187	193	210	216	207	207	198	203
PHV014	PAN	209	209	188	194	179	188	142	154	162	165	160	175	225	234	187	193	210	216	207	207	198	203
PHV015	PAN	209	209	188	194	179	188	142	154	162	165	160	175	225	234	187	193	210	216	207	207	198	203
PHV016	PAN	209	209	188	194	179	188	142	154	162	165	160	175	225	234	187	193	210	216	207	207	198	203
PHV021	PAN	209	209	188	194	179	188	142	154	162	165	160	175	225	234	187	193	210	216	207	207	198	203
PHV022	PAN	209	209	188	194	179	188	142	154	162	165	160	175	225	234	187	193	210	216	207	207	198	203
PHV023	PAN	209	209	188	194	179	188	142	154	162	165	160	175	225	234	187	193	210	216	207	207	198	203
PHV030	PAN	209	209	188	194	179	188	142	154	162	165	160	175	225	234	187	193	210	216	207	207	198	203
PHV034	PAN	209	209	188	194	179	188	142	154	162	165	160	175	225	234	187	193	210	216	207	207	198	203
PHV036	PAN	209	209	188	194	179	188	142	154	162	165	160	175	225	234	187	193	210	216	207	207	198	203
PHV037	PAN	209	209	188	194	179	188	142	154	162	165	160	175	225	234	187	193	210	216	207	207	198	203
PHV039	PAN	209	209	188	194	179	188	142	154	162	165	160	175	225	234	187	193	210	216	207	207	198	203
PHV040	PAN	209	209	188	194	179	188	142	154	162	165	160	175	225	234	187	193	210	216	207	207	198	203
PHV043	PAN	209	209	188	194	179	188	142	154	162	165	160	175	225	234	187	193	210	216	207	207	198	203
PHV045	PAN	209	209	188	194	179	188	142	154	162	165	160	175	225	234	187	193	210	216	207	207	198	203
PHV050	PAN	209	209	188	194	179	188	142	154	162	165	160	175	225	234	187	193	210	216	207	207	198	203
PHV057	PAN	209	209	188	194	179	188	142	154	162	165	160	177	225	234	187	193	210	216	207	207	198	203
PHV058	PAN	209	209	188	194	179	188	142	154	162	165	160	175	225	234	187	193	210	216	207	207	198	203
PHV060	PAN	209	209	188	194	179	188	142	154	162	165	160	175	225	234	187	193	210	216	207	207	198	203
PHV061	PAN	209	209	188	194	179	188	142	154	162	165	160	175	225	234	187	193	210	216	207	207	198	203
PHV062	PAN	209	209	188	194	179	188	142	154	162	165	160	175	225	234	187	193	210	216	207	207	198	203
PHV067	PAN	209	209	188	194	179	188	142	154	162	165	160	175	225	234	187	193	210	216	207	207	198	203
PHV068	PAN	209	209	188	194	179	188	142	154	162	165	160	175	225	234	187	193	210	216	207	207	198	201
PHV070	PAN	209	209	188	194	179	188	142	154	162	165	160	175	225	234	187	193	210	216	207	207	198	203
PHV071	PAN	209	209	188	194	179	188	142	154	162	165	160	175	225	234	187	193	210	216	207	207	198	203
PHV072	PAN	209	209	188	194	179	188	142	154	162	165	160	175	225	234	187	193	210	216	207	207	198	203

PHV073	PAN	209	209	188	194	179	188	142	154	162	165	160	175	225	234	187	193	210	216	207	207	198	203
PHV075	PAN	209	209	188	194	179	188	142	154	162	165	160	175	225	234	187	193	210	216	207	207	198	203
PHV076	PAN	209	209	188	194	179	188	142	154	162	165	160	175	225	234	187	193	210	216	207	207	198	203
PHV077	PAN	209	209	188	194	179	188	142	154	162	165	160	175	225	234	187	193	210	216	207	207	198	203
PHV083	PAN	209	209	188	194	179	188	142	154	162	165	160	175	225	234	187	193	210	216	207	207	198	203
PHV084	PAN	209	209	188	194	179	188	142	154	162	165	160	175	225	234	187	193	210	216	207	207	198	203
PHV090	PAN	209	209	188	194	179	188	142	154	162	165	160	175	225	234	187	193	210	216	207	207	198	203
PHV092	PAN	209	209	188	194	179	188	142	154	162	165	160	175	225	234	187	193	210	216	207	207	198	203
PHV100	PAN	209	209	188	194	179	188	142	154	162	165	160	175	225	234	187	193	210	216	207	207	198	210
PHV104	PAN	209	209	188	194	179	188	142	154	162	165	160	175	225	234	187	193	210	216	207	207	198	203
PHV108	PAN	209	209	188	194	179	188	142	154	162	165	160	175	225	234	187	193	210	216	207	207	198	203
PHV113	PAN	209	209	188	194	179	188	142	154	162	165	160	175	225	234	187	193	210	216	207	207	198	203
PHV114	PAN	209	209	188	194	179	188	142	154	162	165	160	175	225	237	187	193	210	216	207	207	198	203
PHV145	PAN	209	209	188	194	179	188	142	154	162	165	160	175	225	234	187	193	210	216	207	207	198	203
PHV146	PAN	209	209	188	194	179	188	142	154	162	165	160	175	225	234	187	193	210	216	207	219	198	203
PHV147	PAN	209	209	188	194	179	188	142	154	162	165	160	175	225	234	187	193	210	216	207	207	198	203
WCR5	DRC	209	209	188	196	179	188	142	154	165	165	160	175	225	234	187	193	210	216	207	207	198	203
WCR6	DRC	209	209	188	196	179	188	142	154	165	165	160	175	225	234	187	193	210	216	207	207	198	203
WCR7	DRC	209	209	188	196	179	188	142	154	165	165	160	175	225	234	187	193	210	216	207	207	198	203
WCR8	DRC	209	209	188	196	179	188	142	154	165	165	160	175	225	234	187	193	210	216	207	207	198	203
WCR9	DRC	209	209	188	196	179	188	142	154	165	165	160	175	225	234	187	193	210	216	207	207	198	203
WCR10	DRC	209	209	188	196	179	188	142	154	165	165	160	175	225	234	187	193	210	216	207	207	198	203
WCR12	DRC	209	209	188	196	179	188	142	154	165	165	160	175	225	234	187	193	210	216	207	207	198	203
WCR13	DRC	209	209	188	196	179	188	142	154	165	165	160	175	225	234	187	193	210	216	207	207	198	203
WCR15	DRC	209	209	188	196	179	188	142	154	165	165	160	175	225	234	187	193	210	216	207	207	198	203
WCR16	DRC	209	209	188	196	179	188	142	154	165	165	160	175	225	234	187	193	210	216	207	207	198	203
WCR17	DRC	209	209	188	196	179	188	142	154	165	165	160	175	225	234	187	193	210	216	207	207	198	203
WCR18	DRC	209	209	188	196	179	188	142	154	165	165	160	175	225	234	187	193	210	216	207	207	198	203

WCR19	DRC	209	209	188	196	179	188	142	154	165	165	160	175	225	234	187	193	210	216	207	207	198	203
WCR20	DRC	209	209	188	196	179	188	142	154	165	165	160	175	225	234	187	193	210	216	207	207	198	203
WCR21	DRC	209	209	188	196	179	188	142	154	165	165	160	175	225	234	187	193	210	216	207	207	198	203
WCR22	DRC	209	209	188	196	179	194	142	154	165	165	160	175	225	234	187	193	210	216	207	207	198	203
WCR24	DRC	209	209	188	196	179	188	142	154	165	165	160	175	225	234	187	193	210	216	207	207	198	203
WCR25	DRC	209	209	188	196	179	188	142	154	165	165	160	175	225	234	187	193	210	216	207	207	198	203
WCR26	DRC	209	209	188	196	179	188	142	154	165	165	160	175	225	234	187	193	210	216	207	207	198	203
WCR27	DRC	209	209	188	196	179	188	142	154	165	165	160	175	225	234	187	193	210	216	207	207	198	203
WCR28	DRC	209	209	188	196	179	188	142	154	165	165	160	175	225	234	187	193	210	216	207	207	198	203
WCR29	DRC	209	209	188	196	179	188	142	154	165	165	160	175	225	234	187	193	210	216	207	207	198	203
WCR30	DRC	209	209	188	196	179	188	142	154	165	165	160	175	225	234	187	193	210	216	207	207	198	203
WCR-2	COL	209	209	188	194	179	188	142	154	162	165	160	175	225	234	190	193	210	216	207	207	198	203
CO-1	COL	209	211	188	196	179	188	142	154	165	165	160	175	225	234	187	193	210	216	207	207	198	203
CO-2	COL	209	211	188	196	179	188	142	154	165	165	160	175	225	234	187	193	210	216	207	207	198	203
CO-3	COL	209	209	188	196	179	188	142	154	165	165	160	175	225	234	187	193	210	216	207	207	198	203
CO-4	COL	209	211	188	196	179	188	142	154	165	168	160	175	225	234	188	194	210	216	207	207	198	203
CO-5	COL	209	211	188	196	179	188	142	154	165	165	160	175	225	234	187	193	210	216	207	207	198	203
CO-6	COL	209	211	188	196	179	188	142	154	165	165	160	175	225	234	187	193	210	216	207	207	198	203
CO-8	COL	209	211	188	196	179	188	142	154	165	165	160	175	225	234	187	193	210	216	207	207	198	203
CO-9	COL	209	209	188	194	179	188	142	154	162	165	160	175	225	234	190	193	210	216	207	207	198	203
CO-12	COL	209	211	188	196	179	188	142	154	165	165	160	175	225	234	187	193	210	216	207	207	198	203
GT-3	GUAT	209	209	188	194	179	188	142	154	162	165	160	175	225	234	187	193	210	216	207	207	198	203
GT-4	GUAT	209	209	188	194	179	191	142	154	162	165	160	175	225	234	187	193	210	216	207	207	198	203
GT-5	GUAT	209	209	188	194	179	188	142	154	162	165	160	175	225	234	187	193	210	216	207	207	198	203
GT-6	GUAT	209	209	188	194	179	188	142	154	162	165	160	175	225	234	187	193	210	216	207	207	198	203
GT-7	GUAT	209	209	188	194	179	188	142	154	162	165	160	175	225	234	187	193	210	216	207	207	198	203
GT-8	GUAT	209	209	188	194	179	188	142	154	162	165	160	175	225	234	187	193	210	216	207	207	198	203
GT-9	GUAT	209	209	188	194	179	188	142	154	162	165	160	175	225	234	187	193	210	216	207	207	198	203

GT-11	GUAT	209	209	188	194	179	188	142	154	162	165	160	175	225	234	187	193	210	216	207	207	198	203
GT-12	GUAT	209	209	188	194	179	188	142	154	162	165	160	175	225	234	187	193	210	216	207	207	198	203
PUR045839	PERU	209	209	188	194	179	188	142	154	162	165	160	175	225	234	187	193	210	216	207	207	198	203
PUR045840	PERU	209	209	188	194	179	188	142	154	162	165	160	175	225	234	187	193	210	216	207	207	198	203
PUR045841	PERU	209	209	188	194	179	188	142	154	162	165	160	175	225	234	187	193	210	216	207	207	198	203
PUR045842	PERU	209	209	188	194	179	188	142	154	162	165	160	175	225	234	187	193	210	216	207	207	198	203
PUR045844	PERU	209	209	188	194	179	188	142	154	162	165	160	175	225	234	187	193	210	216	207	207	198	203
PUR045845	PERU	209	209	188	194	179	188	142	154	162	165	160	175	225	234	187	193	210	216	207	207	198	203
PUR045848	PERU	209	209	188	194	179	188	142	154	162	165	160	175	225	234	187	193	210	216	207	207	198	203
PUR045843	PERU	209	209	188	194	179	188	142	154	165	165	160	175	225	234	187	193	210	216	207	207	198	203
PURN16756	PERU	209	209	188	194	179	188	142	154	162	165	160	175	225	234	187	193	210	216	207	207	198	203
WCR ES1	ELSL	209	209	188	194	179	188	142	154	165	165	160	175	225	234	187	193	210	216	207	207	198	203
WCR ES2	ELSL	209	209	188	194	179	188	142	154	162	165	160	175	225	234	188	194	210	216	207	207	198	203
WCR ES3	ELSL	209	209	188	194	179	188	142	154	162	165	160	175	225	234	187	193	210	216	207	207	198	203
WCR ES4	ELSL	209	209	188	194	179	188	142	154	162	165	160	175	225	234	187	193	210	216	207	207	198	203
WCR ES5	ELSL	209	209	188	194	179	188	142	154	162	165	160	175	225	234	187	193	210	216	207	207	198	203
WCR ES6	ELSL	209	209	188	194	179	188	142	154	162	165	160	175	225	234	187	193	210	216	207	207	198	203
WCR ES7	ELSL	209	209	188	194	179	188	142	154	162	165	160	175	225	234	187	193	210	216	207	207	198	203
WCR ES8	ELSL	209	209	188	194	179	188	142	154	162	165	160	175	225	234	187	193	210	216	207	207	198	203
WCR ES9	ELSL	209	209	188	194	179	188	142	154	162	168	160	175	225	234	187	193	210	216	207	207	198	203
WCR_TH3	THAI	209	209	188	196	179	188	142	154	162	165	160	175	225	234	187	193	210	216	207	207	198	203
U1618	IDSA	209	209	188	196	179	188	142	154	162	165	160	175	225	237	187	193	210	216	207	207	198	203
PUR018244	IDSA	209	209	188	196	179	188	142	154	162	165	160	175	225	234	187	193	210	216	207	207	198	203
WCR I1	IN	209	209	188	196	179	188	142	154	162	165	160	175	225	237	187	193	210	216	207	207	198	203
WCR I2	IN	209	209	188	194	179	188	142	154	162	165	160	175	225	234	190	193	210	216	207	207	198	203
WCR I3	IN	209	209	188	194	179	188	142	154	162	165	160	175	225	234	187	193	210	216	207	207	198	203
WCR I4	IN	209	209	188	194	179	188	142	154	162	165	150	160	225	234	187	193	210	216	207	207	198	203
WCR I5	IN	209	209	188	194	179	188	142	154	162	165	150	160	225	234	187	193	210	216	207	207	198	201

WCR I6	IN	209	209	188	194	179	188	142	154	162	165	150	160	225	234	187	193	210	216	207	207	198	201
WCR I7	IN	209	209	188	194	179	188	142	154	162	165	150	160	225	234	187	193	210	216	207	207	198	201
WCR I8	IN	209	209	188	194	179	188	142	154	162	165	160	172	225	234	187	193	210	216	207	207	198	203
WCR I10	IN	209	209	188	194	179	188	142	154	162	165	160	172	225	234	187	193	210	216	207	207	198	203
WCR I11	IN	209	209	188	194	179	188	142	154	162	165	160	175	225	234	190	193	210	216	207	207	198	203
WCR I12	IN	209	209	188	194	179	188	142	154	162	165	160	172	225	234	187	193	210	216	207	207	198	203
WCR I13	IN	209	209	188	194	179	191	142	154	162	165	160	175	225	234	190	193	210	216	207	207	198	203
WCR I14	IN	209	209	188	194	179	188	142	154	162	165	150	160	225	234	187	193	210	216	207	207	198	201
WCR I15	IN	209	209	188	194	179	188	142	154	162	165	150	160	225	234	187	193	210	216	207	207	198	201
WCR I16	IN	209	209	188	194	179	188	142	154	162	165	160	172	225	234	187	193	210	216	207	207	198	203
WCR I17	IN	209	209	188	194	179	188	142	154	162	165	160	172	228	234	187	193	210	216	207	207	198	203
U-1540	PR	210	210	188	196	179	188	142	154	165	165	160	175	225	234	187	193	210	216	207	207	198	203
U-1541	PR	209	209	188	194	179	188	142	154	162	165	160	175	225	234	187	193	210	216	207	207	198	203
WCRH1	HOND	209	210	188	194	179	188	142	154	162	165	160	175	225	234	187	193	210	216	207	207	198	203
WCRH2	HOND	209	209	188	194	179	188	142	154	162	165	160	175	225	234	187	193	210	216	207	207	198	203
BA 1-10	JAM	209	209	188	194	179	188	142	154	162	165	160	175	225	234	187	193	210	216	207	207	198	203
BA 1-11	JAM	209	209	188	194	179	188	142	154	162	165	160	175	225	234	187	193	210	216	207	207	198	203
BA 1-12	JAM	209	209	188	194	179	188	142	154	162	165	160	175	225	234	187	193	210	216	207	207	198	203
BA 1-3	JAM	209	209	188	194	179	188	142	154	162	165	160	175	225	234	187	193	210	216	207	207	198	203
BA 1-4	JAM	209	209	188	194	179	188	142	154	162	165	160	175	225	234	187	193	210	216	207	207	198	203
BA 1-5	JAM	209	209	188	194	179	188	142	154	162	165	160	175	225	234	187	193	210	216	207	207	198	203
BA 1-6	JAM	209	209	188	194	179	188	142	154	162	165	160	175	225	234	187	193	210	216	207	207	198	203
BA 1-7	JAM	209	209	188	194	179	188	142	154	162	165	160	175	225	234	187	193	210	216	207	207	198	203
BA 1-8	JAM	209	209	188	194	179	188	142	154	162	165	160	175	225	234	187	193	210	216	207	207	198	203
BA 1-9	JAM	209	209	188	194	179	188	142	154	162	165	160	175	225	234	187	193	210	216	207	207	198	203
BB-4	JAM	209	209	188	194	179	188	142	154	162	165	160	175	225	234	187	193	210	216	207	207	198	203
BB-6	JAM	209	209	188	194	179	188	142	154	162	165	160	175	225	234	187	193	210	216	207	207	198	203
BB-7	JAM	209	209	188	194	179	188	142	154	162	165	160	175	225	234	187	193	210	216	207	207	198	203

BB 1-12	JAM	209	209	188	194	179	188	142	154	162	165	160	175	225	234	187	193	210	216	207	207	198	203
BB 1-9	JAM	209	209	188	194	179	188	142	154	162	165	160	175	225	234	187	193	210	216	207	207	198	203
BC1-11	JAM	209	209	188	194	179	188	142	154	162	165	160	175	225	234	188	194	210	216	207	207	198	203
BC1-12	JAM	209	209	188	194	179	188	142	154	162	165	160	175	225	234	190	194	210	216	207	207	198	203
BC1-2	JAM	209	209	188	194	179	188	142	154	162	165	160	175	225	234	187	194	210	216	207	207	198	203
BC1-3	JAM	209	209	188	194	179	188	142	154	163	166	160	175	225	234	187	193	210	216	207	207	198	203
BC1-4	JAM	209	209	188	194	179	188	142	154	163	166	160	175	225	234	187	193	210	216	207	207	198	203
BC1-6	JAM	209	209	188	194	179	188	142	154	163	166	160	175	225	234	187	193	210	216	207	207	198	203
BC1-7	JAM	209	209	188	194	179	188	142	154	162	165	160	175	225	234	187	193	210	216	207	207	198	203
BC1-8	JAM	209	209	188	194	179	188	142	154	162	165	160	175	225	234	188	194	210	216	207	207	198	203
BC10	JAM	209	209	188	194	179	188	142	154	162	165	160	175	225	234	188	194	210	216	207	207	198	203
BC11	JAM	209	209	188	194	179	188	142	154	162	165	160	175	225	234	188	194	210	216	207	207	198	203
BC12	JAM	209	209	188	194	179	188	142	154	162	165	160	175	225	234	188	194	210	216	207	207	198	203
BC3	JAM	209	209	188	194	179	188	142	154	162	165	160	175	225	234	188	194	210	216	207	207	198	203
BC4	JAM	209	209	188	194	179	188	142	154	162	165	160	175	225	234	188	194	210	216	207	207	198	203
BC5	JAM	209	209	188	194	179	188	142	154	162	165	160	175	225	234	188	194	210	216	207	207	198	203
BC6	JAM	209	209	188	194	179	188	142	154	162	165	160	175	225	234	188	194	210	216	207	207	198	203
BC7	JAM	209	209	188	194	179	188	142	154	162	165	160	175	225	234	188	194	210	216	207	207	198	203
BC8	JAM	209	209	188	194	179	188	142	154	162	165	160	175	225	234	188	194	210	216	207	207	198	203
CE-11	JAM	209	209	188	194	179	188	142	154	162	165	160	175	225	234	187	193	210	216	207	207	198	203
CE-2	JAM	209	209	188	194	179	188	142	154	162	165	160	175	225	234	187	193	210	216	207	207	198	203
CE-4	JAM	209	209	188	194	179	188	142	154	162	165	160	175	225	234	187	193	210	216	207	207	198	203
CE-5	JAM	209	209	188	194	179	188	142	154	162	165	160	175	225	234	187	193	210	216	207	207	198	203
CE-6	JAM	209	209	188	194	179	188	142	154	162	165	160	175	225	234	187	193	210	216	207	207	198	203
CE-9	JAM	209	209	188	194	179	188	142	154	162	165	160	175	225	234	187	193	210	216	207	207	198	203
CE1-1	JAM	209	209	188	194	179	188	142	154	162	165	160	175	225	234	187	193	210	216	207	207	198	203
CE1-11	JAM	209	209	188	194	179	188	142	154	162	165	160	175	225	234	187	193	210	216	207	207	198	203
CE1-13	JAM	209	209	188	194	179	188	142	154	162	165	160	175	225	234	187	193	210	216	207	207	198	203

CE1-14	JAM	209	209	188	194	179	188	142	154	162	165	160	175	225	234	187	193	210	216	207	207	198	203
CE1-15	JAM	209	209	188	194	179	188	142	154	162	165	160	175	225	234	187	193	210	216	207	207	198	203
CE1-3	JAM	209	209	188	194	179	188	142	154	162	165	160	175	225	234	187	193	210	216	207	207	198	203
CE1-4	JAM	209	209	188	194	179	188	142	154	162	165	160	175	225	234	187	193	210	216	207	207	198	203
CE1-5	JAM	209	209	188	194	179	188	142	154	162	165	160	175	225	234	187	193	210	216	207	207	198	203
CE1-6	JAM	209	209	188	194	179	188	142	154	162	165	160	175	225	234	187	193	210	216	207	207	198	203
CE1-7	JAM	209	209	188	194	179	188	142	154	162	165	160	175	225	234	187	193	210	216	207	207	198	203
CE1-8	JAM	209	209	188	194	179	188	142	154	162	165	160	175	225	234	187	193	210	216	207	207	198	203
CE1-9	JAM	209	209	188	194	179	188	142	154	162	165	160	175	225	234	187	193	210	216	207	207	198	203
DW-10	JAM	209	209	188	194	179	188	142	154	162	165	160	175	225	234	187	193	210	216	207	207	198	203
DW-12	JAM	209	209	188	194	179	188	142	154	162	165	160	175	225	234	187	193	210	216	207	207	198	203
DW-4	JAM	209	209	188	194	179	188	142	154	162	165	160	175	225	234	187	193	210	216	207	207	198	203
DW-5	JAM	209	209	188	194	179	188	142	154	162	165	160	175	225	234	187	193	210	216	207	207	198	203
DW-7	JAM	209	209	188	194	179	188	142	154	162	165	160	175	225	234	187	193	210	216	207	207	198	203
DW-9	JAM	209	209	188	194	179	188	142	154	162	165	160	175	225	234	187	193	210	216	207	207	198	203
DW1-11	JAM	209	209	188	194	179	188	142	154	162	165	160	175	225	234	187	193	210	216	207	207	198	203
DW1-3	JAM	209	209	188	194	179	188	142	154	162	165	160	175	225	234	187	193	210	216	207	207	198	203
DW1-5	JAM	209	209	188	194	179	188	142	154	162	165	160	175	225	234	187	193	210	216	207	207	198	203
DW1-7	JAM	209	209	188	194	179	188	142	154	162	165	160	175	225	234	187	193	210	216	207	207	198	198
DW1-9	JAM	209	209	188	194	179	188	142	154	162	165	160	175	225	234	187	193	210	216	207	207	198	203
EL-10	JAM	209	209	188	194	179	188	142	154	162	165	160	175	225	234	187	193	210	216	207	207	198	203
EL-11	JAM	209	209	188	194	179	188	142	154	162	165	160	175	225	234	187	193	210	216	207	207	198	203
EL-3	JAM	209	209	188	194	179	188	142	154	162	165	160	175	225	234	188	194	210	216	207	207	198	203
EL-5	JAM	209	209	188	194	179	188	142	154	162	165	160	175	225	234	187	193	210	216	207	207	198	203
EL-6	JAM	209	209	188	194	179	188	142	154	162	165	160	175	225	234	187	193	210	216	207	207	198	203
EL-8	JAM	209	209	188	194	179	188	142	154	162	165	160	175	225	234	188	194	210	216	207	207	198	203
EL-9	JAM	209	209	188	194	179	188	142	154	162	165	160	175	225	234	188	194	210	216	207	207	198	203
EL1-5	JAM	209	209	188	194	179	188	142	154	162	165	160	175	225	234	187	196	210	216	207	207	198	203

EL1-7	JAM	209	209	188	194	179	188	142	154	162	165	160	175	225	234	187	196	210	216	207	207	198	203
EL1-8	JAM	209	209	188	194	179	188	142	154	162	165	160	175	225	234	187	193	210	216	207	207	198	203
EL1-9	JAM	209	209	188	194	179	188	142	154	162	165	160	175	225	234	187	193	210	216	207	207	198	203
GW-10	JAM	209	209	188	194	179	188	142	154	162	165	160	175	225	234	187	193	210	216	207	207	198	203
GW-12	JAM	209	209	188	194	179	188	142	154	162	165	160	175	225	234	188	194	210	216	207	207	198	203
GW-3	JAM	209	209	188	194	179	188	142	154	162	165	160	175	225	234	188	194	210	216	207	207	198	203
GW-4	JAM	209	209	188	194	179	188	142	154	162	165	160	175	225	234	187	193	210	216	207	207	198	203
GW-5	JAM	209	209	188	194	179	188	142	154	162	165	160	175	225	234	188	194	210	216	207	207	198	203
GW-7	JAM	209	209	188	194	179	188	142	154	162	165	160	175	225	234	188	194	210	216	207	207	198	203
GW-8	JAM	209	209	188	194	179	188	142	154	162	165	160	175	225	234	188	194	210	216	207	207	198	203
GW-9	JAM	209	209	188	194	179	188	142	154	162	165	160	175	225	234	188	194	210	216	207	207	198	203
GW1-2	JAM	209	209	188	194	179	188	142	154	162	165	160	175	225	234	187	193	210	216	207	207	198	203
GW1-3	JAM	209	209	188	194	179	188	142	154	162	165	160	175	225	234	187	193	210	216	207	207	198	203
GW1-5	JAM	209	209	188	194	179	188	142	154	162	165	160	175	225	234	187	193	210	216	207	207	198	203
GW1-6	JAM	209	209	188	194	179	188	142	154	162	165	160	175	225	234	187	193	210	216	207	207	198	203
GW1-9	JAM	209	209	188	194	179	188	142	154	162	165	160	175	225	234	187	193	210	216	207	207	198	203
HV-10	JAM	209	209	188	196	179	188	142	154	165	165	160	175	225	234	187	193	210	216	207	207	198	203
HV-3	JAM	209	209	188	196	179	188	142	154	165	165	160	175	225	234	187	193	210	216	207	207	198	203
HV-4	JAM	209	209	188	194	179	188	142	154	162	165	160	175	225	234	187	193	210	216	207	207	145	198
HV-5	JAM	209	209	188	194	179	188	142	154	162	165	160	175	225	234	187	193	210	216	207	207	198	203
HV-7	JAM	209	209	188	194	179	188	142	154	162	165	160	175	225	234	187	193	210	216	207	207	198	203
HV-8	JAM	209	209	188	194	179	188	142	154	162	165	160	175	225	234	187	193	210	216	207	207	198	203
HV-9	JAM	209	209	188	194	179	188	142	154	160	165	160	175	225	234	187	193	210	216	207	207	198	203
HV1-10	JAM	209	209	188	194	179	188	142	154	162	165	160	175	225	234	187	193	210	216	207	207	198	203
HV1-12	JAM	209	209	188	194	179	188	142	154	160	165	160	175	225	234	187	193	210	216	207	207	198	203
HV1-2	JAM	209	209	188	194	179	188	142	154	162	165	160	175	225	234	187	193	210	216	207	207	198	203
HV1-7	JAM	209	209	188	194	179	188	142	154	162	165	160	175	225	234	187	193	210	216	207	207	198	203
HV1-8	JAM	209	209	188	194	179	188	142	154	162	165	160	175	225	234	187	193	210	216	207	207	198	203

KP-10	JAM	209	209	188	194	179	188	142	154	162	165	160	175	225	234	188	194	210	216	207	207	198	203
KP-11	JAM	209	209	188	194	179	188	142	154	162	165	160	175	225	234	187	193	210	216	207	207	198	203
KP-12	JAM	209	209	188	194	179	188	142	154	162	165	160	175	225	234	188	194	210	216	207	207	198	203
KP-2	JAM	209	209	188	194	179	188	142	154	162	165	160	175	225	234	188	194	210	216	207	207	198	203
KP-3	JAM	209	209	188	194	179	188	142	154	162	165	160	175	225	234	188	194	210	216	207	207	198	203
KP-4	JAM	209	209	188	194	179	188	142	154	162	165	160	175	225	234	188	194	210	216	207	207	198	203
KP-6	JAM	209	209	188	194	179	188	142	154	162	165	160	175	225	234	188	194	210	216	207	207	199	203
KP-7	JAM	209	209	188	194	179	188	142	154	162	165	160	175	225	234	187	193	210	216	207	207	198	203
KP-8	JAM	209	209	188	194	179	188	142	154	162	165	160	175	225	234	187	193	210	216	207	207	198	203
KP1-1	JAM	209	209	188	194	179	188	142	154	162	165	160	175	225	234	187	193	210	216	207	207	198	203
KP1-10	JAM	209	209	188	194	179	188	142	154	162	165	160	175	225	234	188	193	210	216	207	207	198	203
KP1-11	JAM	209	209	188	194	179	188	142	154	162	165	160	175	225	234	187	193	210	216	207	207	198	203
KP1-12	JAM	209	209	188	194	179	188	142	154	162	165	160	175	225	234	188	194	210	216	207	207	198	203
KP1-2	JAM	209	209	188	194	179	188	142	154	162	165	160	175	225	234	187	193	210	216	207	207	198	203
KP1-3	JAM	209	209	188	194	179	188	142	154	162	165	160	175	225	234	187	193	210	216	207	207	198	203
KP1-4	JAM	209	209	188	194	179	188	142	154	162	165	160	175	225	234	187	193	210	216	207	207	198	203
KP1-5	JAM	209	209	188	194	179	188	142	154	163	166	160	175	225	234	187	193	210	216	207	207	198	203
KP1-6	JAM	209	209	188	194	179	188	142	154	163	165	160	175	225	234	187	193	210	216	207	207	198	203
KP1-7	JAM	209	209	188	194	179	188	142	154	162	165	160	175	225	234	188	194	210	216	207	207	198	203
KP1-8	JAM	209	209	188	194	179	188	142	154	162	165	160	175	225	234	187	193	210	216	207	207	198	203
KP1-9	JAM	209	209	188	194	179	188	142	154	162	165	160	175	225	234	187	193	210	216	207	207	198	203
MB-10	JAM	209	209	188	194	179	188	142	154	162	165	160	175	225	234	187	193	210	216	207	207	198	203
MB-11	JAM	209	209	188	194	179	188	142	154	162	165	160	175	225	234	187	193	210	216	207	207	198	203
MB-2	JAM	209	209	188	194	179	188	142	154	162	165	160	175	225	234	187	193	210	216	207	207	198	203
MB-3	JAM	209	209	188	194	179	188	142	154	162	165	160	175	225	234	187	193	210	216	207	207	198	203
MB-5	JAM	209	209	188	194	179	188	142	154	162	165	160	175	225	234	187	193	210	216	207	207	198	203
MB-6	JAM	209	209	188	194	179	188	142	154	162	165	160	175	225	234	187	193	210	216	207	207	198	203
MB-7	JAM	209	209	188	194	179	188	142	154	162	165	160	175	225	234	187	193	210	216	207	207	198	203

MB-8	JAM	209	209	188	194	179	188	142	154	162	165	160	175	225	234	187	193	210	216	207	207	198	203
MB-9	JAM	209	209	188	194	179	188	142	154	162	165	160	175	225	234	187	193	210	216	207	207	198	203
MB1-10	JAM	209	209	188	194	179	188	142	154	162	165	160	175	225	234	187	193	210	216	207	207	198	203
MB1-12	JAM	209	209	188	194	179	188	142	154	162	165	160	175	225	234	187	193	210	216	207	207	198	203
MB1-2	JAM	209	209	188	194	179	188	142	154	162	165	160	175	225	234	187	193	210	216	207	207	198	203
MB1-4	JAM	209	209	188	194	179	188	142	154	162	165	160	175	225	234	187	193	210	216	207	207	198	203
MB1-5	JAM	209	209	188	194	179	188	142	154	162	165	160	175	225	234	187	193	210	216	207	207	198	203
MB1-8	JAM	209	209	188	194	179	188	142	154	162	165	160	175	225	234	187	193	210	216	207	207	198	203
MB1-9	JAM	209	209	188	194	179	188	142	154	162	165	160	175	225	234	187	193	210	216	207	207	198	203
MF-10	JAM	209	209	188	194	179	188	142	154	162	165	160	175	225	234	187	193	210	216	207	207	198	203
MF-11	JAM	209	209	188	194	179	188	142	154	162	165	160	175	225	234	187	193	210	216	207	207	198	203
MF-2	JAM	209	209	188	194	179	188	142	154	162	165	160	175	225	234	187	193	210	216	207	207	198	203
MF-4	JAM	209	209	188	194	179	188	142	154	162	165	160	175	225	234	187	193	210	216	207	207	198	203
MF-5	JAM	209	209	188	194	179	188	142	154	162	165	160	175	225	234	187	193	210	216	207	207	198	203
MF-7	JAM	209	209	188	194	179	188	142	154	162	165	160	175	225	234	187	193	210	216	207	207	198	203
MF-8	JAM	209	209	188	194	179	188	142	154	162	165	160	175	225	234	187	193	210	216	207	207	198	203
MF-9	JAM	209	209	188	194	179	188	142	154	162	165	160	175	225	234	187	193	210	216	207	207	198	203
MF1-10	JAM	209	209	188	194	179	188	142	154	162	165	160	175	225	234	188	194	210	216	207	207	198	203
MF1-11	JAM	209	209	188	194	179	188	142	154	162	165	160	175	206	225	188	194	210	216	207	207	198	203
MF1-5	JAM	209	209	188	194	179	188	142	154	162	165	160	175	225	234	187	193	210	216	207	207	198	203
MF1-6	JAM	209	209	188	194	179	188	142	154	162	165	160	175	225	234	187	193	210	216	207	207	198	203
MF1-7	JAM	209	209	188	194	179	188	142	154	162	165	160	175	225	234	188	194	210	216	207	207	198	203
MH1-11	JAM	209	209	188	194	179	188	142	154	162	165	160	175	225	234	187	193	210	216	207	207	198	203
MH1-2	JAM	209	209	188	194	179	188	142	154	162	165	160	175	225	234	187	193	210	216	207	207	198	203
MH1-4	JAM	209	209	188	194	179	188	142	154	162	165	160	175	225	234	187	193	210	216	207	207	198	203
MH1-6	JAM	209	209	188	194	179	188	142	154	162	165	160	175	225	234	187	193	210	216	207	207	198	203
MH1-7	JAM	209	209	188	194	179	188	142	154	162	165	160	175	225	234	187	193	210	216	207	207	198	203
MH1-8	JAM	209	209	188	194	179	188	142	154	162	165	160	175	225	234	187	193	210	216	207	207	198	203

MH1-9	JAM	209	209	188	194	179	188	142	154	162	165	160	175	225	234	187	193	210	216	207	207	198	203
PH-10	JAM	209	209	188	194	179	188	142	154	162	165	160	175	225	234	187	193	210	216	207	207	198	203
PH-11	JAM	209	209	188	194	179	188	142	154	162	165	160	175	225	234	187	193	210	216	207	207	198	203
PH-12	JAM	209	209	188	194	179	188	142	154	162	165	160	175	225	234	187	193	210	216	207	207	198	203
PH-2	JAM	209	209	188	194	179	188	142	154	162	165	160	175	225	234	187	193	210	216	207	207	198	203
PH-3	JAM	209	209	188	196	179	188	142	154	162	165	160	175	225	234	187	193	210	216	207	207	198	203
PH-4	JAM	209	209	188	194	179	188	142	154	162	165	160	175	225	234	187	193	210	216	207	207	198	203
PH-6	JAM	209	209	188	194	179	188	142	154	162	165	160	175	225	234	187	193	210	216	207	207	198	203
PH-9	JAM	209	209	188	194	179	188	142	154	162	165	160	175	225	234	187	193	210	216	207	207	198	203
PH1-11	JAM	209	209	188	196	179	188	142	154	162	165	160	175	225	234	187	193	210	216	207	207	198	203
PH1-12	JAM	209	209	188	194	179	188	142	154	162	165	160	175	225	234	187	193	210	216	207	207	198	203
PH1-2	JAM	209	209	188	196	179	188	142	154	162	165	160	175	225	234	187	193	210	216	207	207	198	203
PH1-5	JAM	209	209	188	196	179	188	142	154	162	165	160	175	225	234	187	193	210	216	207	207	198	203
PH1-6	JAM	209	209	188	196	179	188	142	154	162	165	160	175	225	234	187	193	210	216	207	207	198	203
PH1-7	JAM	209	209	188	196	179	188	142	154	162	165	160	175	225	234	187	193	210	216	207	207	198	203
PH1-8	JAM	209	209	188	196	179	188	142	154	162	165	160	175	225	234	187	193	210	216	207	207	198	203
PH1-9	JAM	209	209	188	196	179	188	142	154	162	165	160	175	225	234	187	193	210	216	207	207	198	203
SJ1-1	JAM	209	209	188	194	179	188	142	154	162	165	160	175	225	234	187	193	210	216	207	207	198	203
SJ1-6	JAM	209	209	188	194	179	188	142	154	162	165	160	175	225	234	187	193	210	216	207	207	198	203
TP-11	JAM	209	209	188	194	179	188	142	154	162	165	160	175	225	234	187	193	210	216	207	207	198	203
TP-3	JAM	209	209	188	194	179	188	142	154	162	165	160	175	225	234	187	193	210	216	207	207	198	203
TP-4	JAM	209	209	188	194	179	188	142	154	162	165	160	175	225	234	187	193	210	216	207	207	198	203
TP-8	JAM	209	209	188	194	179	188	142	154	162	165	160	175	225	234	187	193	210	216	207	207	198	203
TP-9	JAM	209	209	188	194	179	188	142	154	162	165	160	175	225	234	187	193	210	216	207	207	198	203
TP1-1	JAM	209	209	188	194	179	188	142	154	162	165	160	175	225	234	187	193	210	216	207	207	198	203
TP1-12	JAM	209	209	188	194	179	188	142	154	162	165	160	175	225	234	187	193	210	216	207	207	198	203
TQ-10	JAM	209	209	188	194	179	188	142	154	162	165	160	175	225	234	187	193	210	216	207	207	198	203
TQ-11	JAM	209	209	188	194	179	188	142	154	162	165	160	175	225	234	187	193	210	216	207	207	198	203

TQ-12	JAM	209	209	188	194	179	188	142	154	162	165	160	175	225	234	187	193	210	216	176	207	198	203
TQ-2	JAM	209	209	188	194	179	188	142	154	162	165	160	175	225	234	187	193	210	216	207	207	198	203
TQ-3	JAM	209	209	188	194	179	188	142	154	162	165	160	175	225	234	187	193	210	216	207	207	198	203
TQ-4	JAM	209	209	188	194	179	188	142	154	162	165	160	175	225	234	187	193	210	216	207	207	198	203
TQ-5	JAM	209	209	188	194	179	188	142	154	162	165	160	175	225	234	187	193	210	216	207	207	198	203
TQ-6	JAM	209	209	188	194	179	188	142	154	162	165	160	175	225	234	187	193	210	216	207	207	198	203
TQ-7	JAM	209	209	188	194	179	188	142	154	162	165	160	175	225	234	187	193	210	216	207	207	198	203
TQ-8	JAM	209	209	188	194	179	188	142	154	162	165	160	175	225	234	187	193	210	216	207	207	198	203
TQ1-4	JAM	209	209	188	194	179	188	142	154	162	165	160	175	225	234	187	193	210	216	207	207	198	203
TQ1-6	JAM	209	209	188	194	179	188	142	154	162	165	160	175	225	234	187	193	210	216	207	207	198	203
TQ1-7	JAM	209	209	188	194	179	188	142	154	162	165	160	175	225	234	187	193	210	216	207	207	198	203
TQ1-9	JAM	209	209	188	194	179	188	142	154	162	165	160	175	225	234	187	193	210	216	207	207	198	203
WF-10	JAM	209	209	188	194	179	188	142	154	162	165	160	175	225	234	188	194	210	216	207	207	198	203
WF-11	JAM	209	209	188	194	179	188	142	154	162	165	160	175	225	234	188	194	210	216	207	207	198	203
WF-2	JAM	209	209	188	194	179	188	142	154	162	165	160	175	225	234	188	194	210	216	207	207	198	203
WF-3	JAM	209	209	188	194	179	188	142	154	162	165	160	175	225	234	188	194	210	216	207	207	198	203
WF-4	JAM	209	209	188	194	179	188	142	154	162	165	160	175	225	234	188	194	210	216	207	207	198	203
WF-5	JAM	209	209	188	194	179	188	142	154	162	165	160	175	225	234	187	193	210	216	207	207	198	203
WF-7	JAM	209	209	188	194	179	188	142	154	162	165	160	175	225	234	187	193	210	216	207	207	198	203
WF-9	JAM	209	209	188	194	179	188	142	154	162	165	160	175	225	234	188	193	210	216	207	207	198	203
WF1-10	JAM	209	209	188	194	179	188	142	154	162	165	160	175	225	234	187	193	210	216	207	207	198	203
WF1-11	JAM	209	209	188	194	179	188	142	154	162	165	160	175	225	234	188	194	210	216	207	207	198	203
WF1-12	JAM	209	209	188	194	179	188	142	154	162	165	160	175	225	234	188	194	210	216	207	207	198	203
WF1-4	JAM	209	209	188	194	179	188	142	154	162	165	160	175	225	234	187	193	210	216	207	207	198	203
WF1-6	JAM	209	209	188	194	179	188	142	154	162	165	160	175	225	234	187	193	210	216	207	207	198	203
WF1-7	JAM	209	209	188	194	179	188	142	154	162	165	160	175	225	234	187	193	210	216	207	207	198	203
WF1-8	JAM	209	209	188	194	179	188	142	154	162	165	160	175	225	234	187	193	210	216	207	207	198	203
WF1-9	JAM	209	209	188	194	179	188	142	154	162	165	160	175	225	234	187	193	210	216	207	207	198	203

BA 2 (A)	JAM	209	209	188	194	179	188	142	154	162	165	160	175	225	234	187	193	210	216	207	207	198	203
BB-6	JAM	209	209	188	194	179	188	142	154	162	165	160	175	225	234	188	194	210	216	207	207	198	203
BB-7 (A)	JAM	209	209	188	194	179	188	142	154	162	165	160	175	225	234	187	193	210	216	207	207	198	203
DW-3 (B)	JAM	209	209	188	194	179	188	142	154	162	165	160	175	225	234	187	193	210	216	207	207	198	203
DW-8 (A)	JAM	209	209	188	194	179	188	142	154	162	165	160	175	225	234	187	193	210	216	207	207	198	203
EL-10	JAM	209	209	188	194	179	188	142	154	162	165	160	175	225	234	187	194	210	216	207	207	198	203
EL-11 (A)	JAM	209	209	188	194	179	188	142	154	162	165	160	175	225	234	187	194	210	216	207	207	198	203
HV-5	JAM	209	209	188	194	179	188	142	154	162	165	160	175	225	234	187	193	210	216	207	207	198	203
MF-6	JAM	209	209	188	194	179	188	142	154	162	165	160	175	225	234	187	193	210	216	207	207	198	203
MH-12	JAM	209	209	188	194	179	188	142	154	162	165	160	175	225	234	187	193	210	216	207	207	198	203
TP-6 (B)	JAM	209	209	188	194	179	188	142	154	162	165	160	175	225	234	187	193	210	216	207	207	198	203
TQ-1	JAM	209	209	188	194	179	188	142	154	162	165	160	175	225	234	187	193	210	216	207	207	198	203
TQ-3	JAM	209	209	188	194	179	188	142	154	162	165	160	175	225	234	187	193	210	216	207	207	198	203
TQ-8	JAM	209	209	188	194	179	188	142	154	162	165	160	175	225	234	187	193	210	216	207	207	198	203
U1771 a	HI	209	209	188	194	180	189	142	154	162	165	160	175	225	234	187	193	210	216	207	207	198	203
U1771 b	HI	209	209	188	194	180	189	142	154	162	165	160	175	225	234	187	193	210	216	207	207	198	203
U1772 a	HI	209	209	188	194	179	188	142	154	162	165	160	175	225	234	187	193	210	216	207	207	198	203
U1772 b	HI	209	209	188	194	180	189	142	154	162	165	160	175	225	234	187	193	210	216	207	207	198	203
U1773 a	HI	209	209	188	194	179	188	142	154	162	165	160	175	225	234	187	193	210	216	207	207	198	203
U1773 b	HI	209	209	188	194	179	188	142	154	162	165	160	175	225	234	187	193	210	216	207	207	198	203
U1774 A	HI	209	209	188	194	180	189	142	154	162	165	160	175	225	234	187	193	210	216	207	207	198	203
U1774 B	HI	209	209	188	194	180	189	142	154	162	165	160	175	225	234	187	193	210	216	207	207	198	203
U1774 C	HI	209	209	188	194	180	189	142	154	162	165	160	175	225	234	187	193	210	216	207	207	198	203
U1774 D	HI	209	209	188	194	180	189	142	154	162	165	160	175	225	234	187	193	210	216	207	207	198	203
PUR018142	BRZL	209	211	188	196	179	188	142	154	165	165	160	175	225	234	187	193	210	216	207	207	198	203
PUR018143	BRZL	209	209	188	196	179	188	142	154	165	165	160	175	225	234	187	193	210	216	207	207	198	203
PUR018146	BRZL	209	209	188	196	179	188	142	154	165	165	160	175	225	234	187	193	210	216	207	207	198	203
PUR018147	BRZL	209	209	188	194	179	188	142	154	165	165	160	175	225	234	187	193	210	216	207	207	198	203

PUR018150	BRZL	209	209	188	196	179	188	142	154	165	165	160	175	225	234	187	193	210	216	207	207	198	203
PUR018151	BRZL	209	209	188	196	179	188	142	154	165	165	160	175	225	234	187	193	210	216	207	207	198	203
PUR018152	BRZL	209	211	188	196	179	188	142	154	165	165	160	175	225	234	187	193	210	216	207	207	198	203
PUR018155	BRZL	209	209	188	196	179	188	142	154	165	165	160	175	225	234	187	193	210	216	207	207	198	201
PUR018158	BRZL	209	209	188	196	179	188	142	154	165	165	160	175	225	234	187	193	210	216	207	207	198	203
PUR018163	BRZL	209	209	188	196	179	188	142	154	165	165	160	175	225	234	187	193	210	216	207	207	198	203
PUR018165	BRZL	209	209	188	196	179	188	142	154	165	165	160	175	225	234	187	193	210	216	207	207	198	203
PUR018167	BRZL	209	209	188	196	179	188	142	154	165	165	160	175	225	234	187	193	210	216	207	207	198	203
PUR018168	BRZL	209	211	188	196	179	188	142	154	165	165	160	175	225	234	187	193	210	216	207	207	198	203
PUR018170	BRZL	209	211	188	196	179	188	142	154	165	165	160	175	225	234	187	193	210	216	207	207	198	203
PUR018171	BRZL	209	211	188	196	179	188	142	154	165	165	160	175	225	234	187	193	210	216	207	207	198	203
PUR018172	BRZL	209	209	188	196	179	188	142	154	165	165	160	175	225	234	187	193	210	216	207	207	198	203
PUR018175	BRZL	209	209	188	196	179	188	142	154	165	165	160	175	225	234	187	193	210	216	207	207	198	203
PUR018178	BRZL	209	209	188	196	179	188	142	154	165	165	160	175	225	234	187	193	210	214	207	207	198	203
PUR018179	MM	209	209	188	196	179	188	142	154	165	165	160	175	225	234	187	193	210	216	207	207	198	203
PUR018237	NRA	211	211	188	196	179	188	142	154	165	165	160	175	225	234	190	193	210	216	207	207	198	203

[a] **Ind,** individuals; [b] **Pop,** population distributed as follow: ETH, Ethiopia; CMRN, Cameroon; PAN, Panama; DRC, Democratic Republic of the Congo; COL, Colombia; GUAT, Guatemala; PERU, Peru; ELSL, El Salvador; THAI, Thailand; IDSA, Indonesia; IN, India; PR, Puerto Rico; HOND, Honduras; JAM, Jamaica; HI, Hawaii; BRZL, Brazil; MM, Myanmar; NRA, Nigeria.

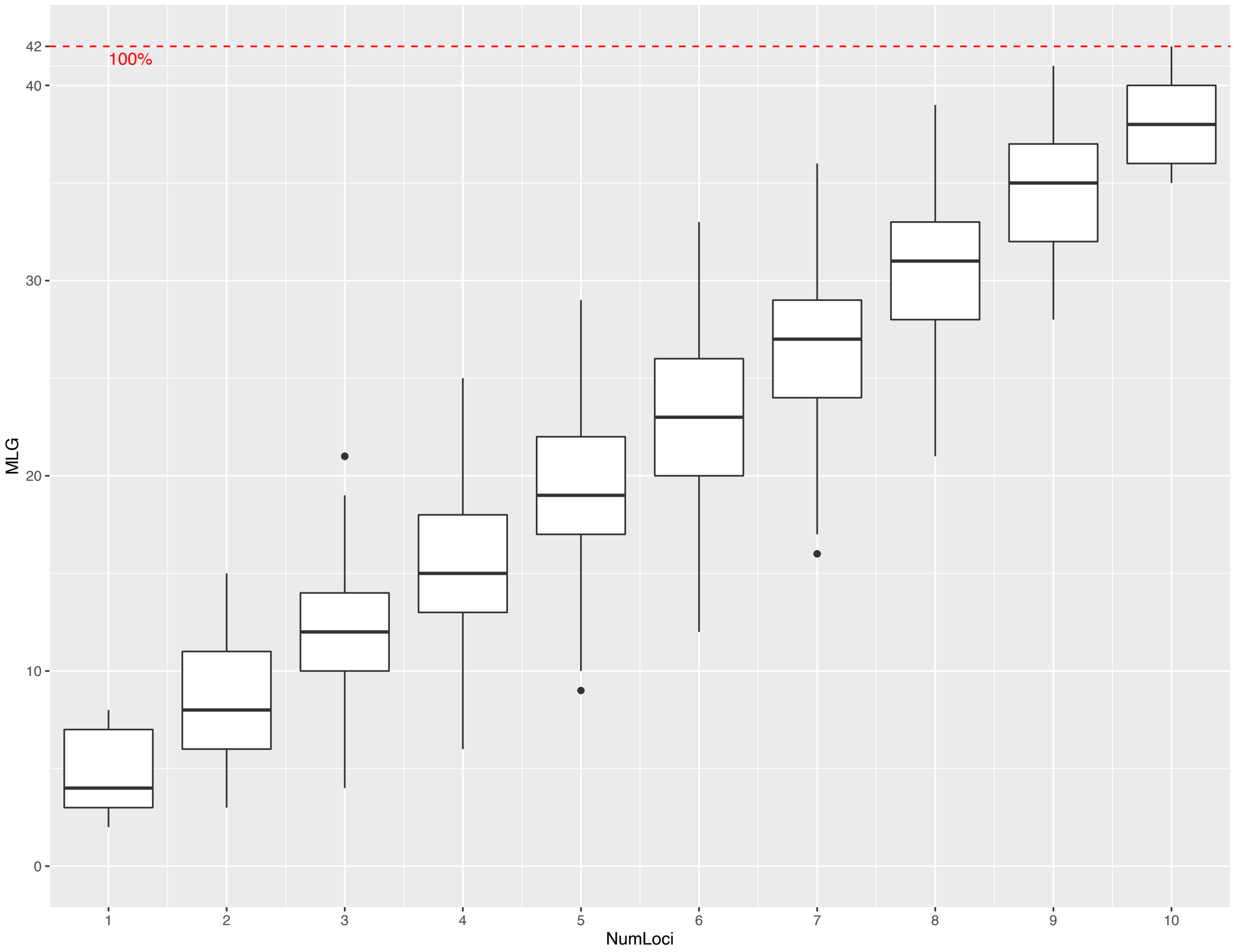

Figure S1. Genotype accumulation curve for eleven SSR markers characterized in 434 *Hemileia vastatrix* specimens from global population. The horizontal axis represents the number of loci randomly sampled up to $n - 1$ loci (10), the vertical axis shows the number of multilocus genotypes observed (42). Boxplots represent random allele sampling with replacement (n = 1000) at each locus. The red dashed line denotes 100% of the total multilocus genotypes identified in the dataset.

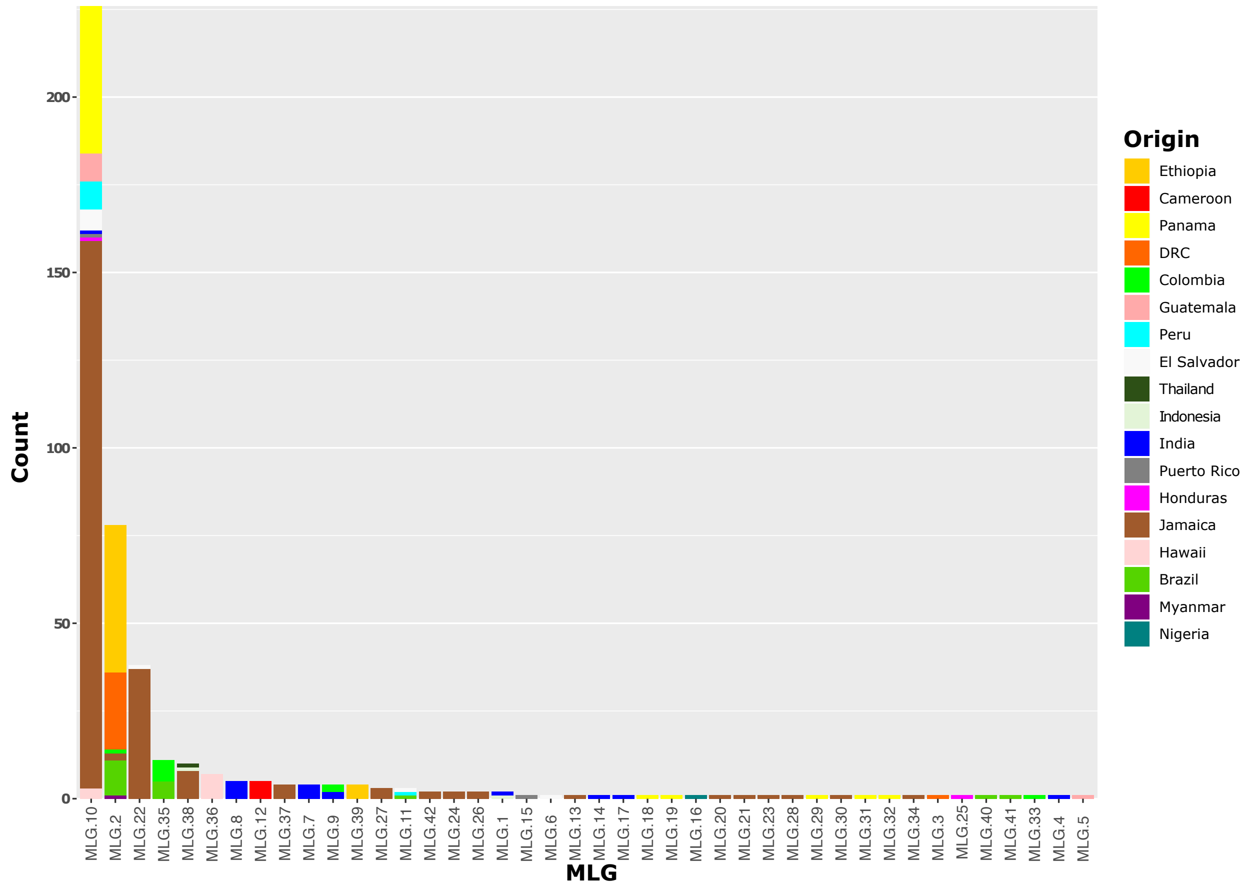

Figure S2. Distribution of genotypic diversity in 434 specimens of *Hemileia vastatrix*. Column bars denote abundance of MLGs with countries represented with colors. **MLG**, number of multilocus genotypes observed.

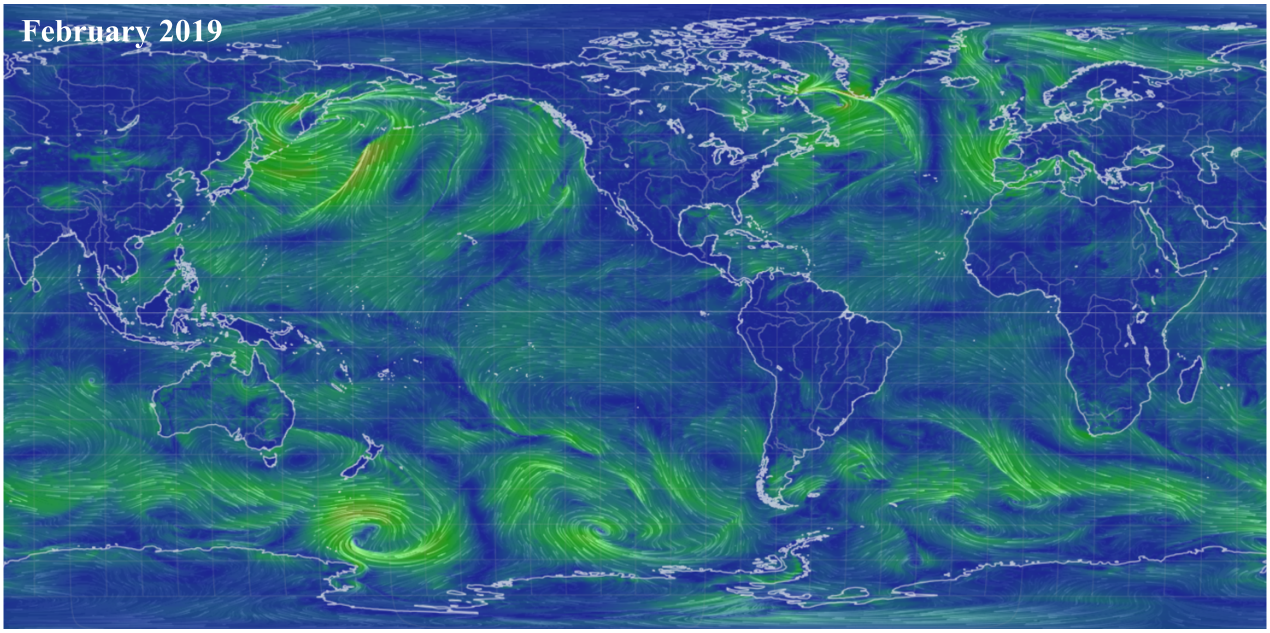
February 2019

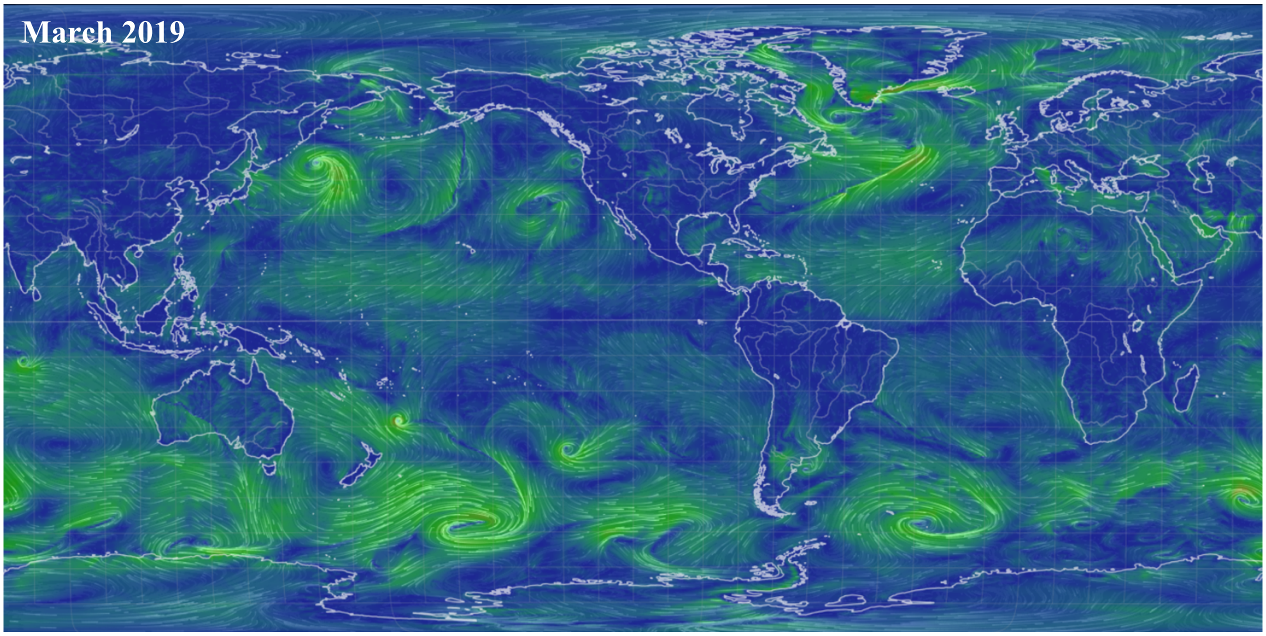
March 2019

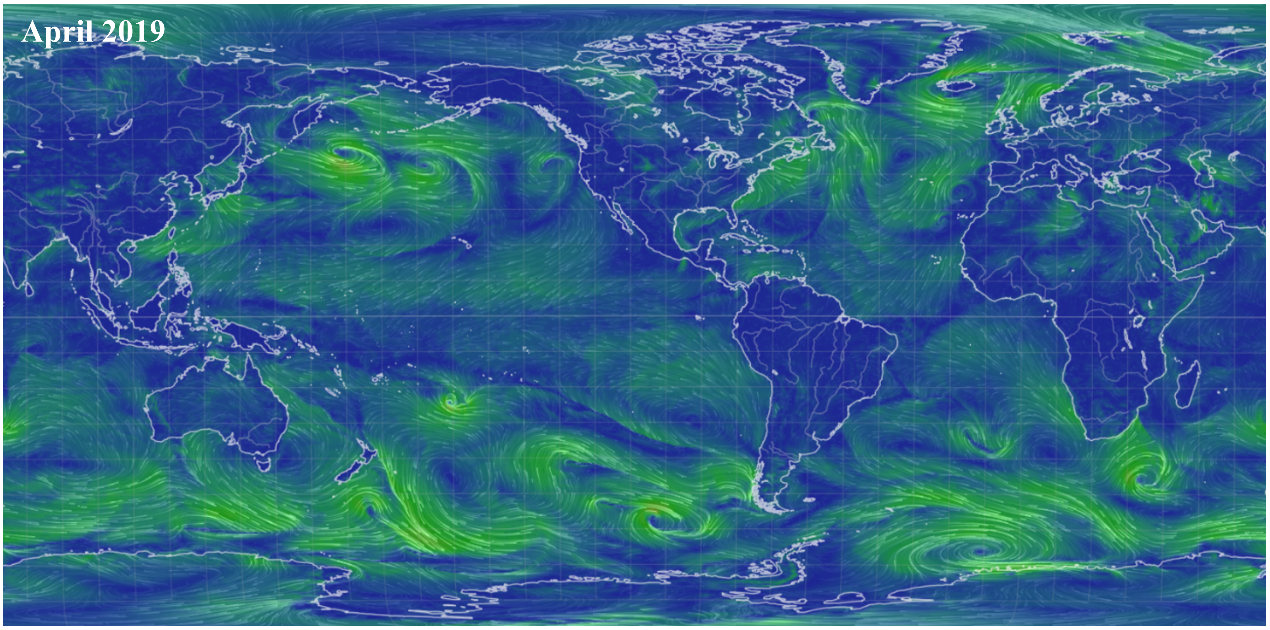
April 2019

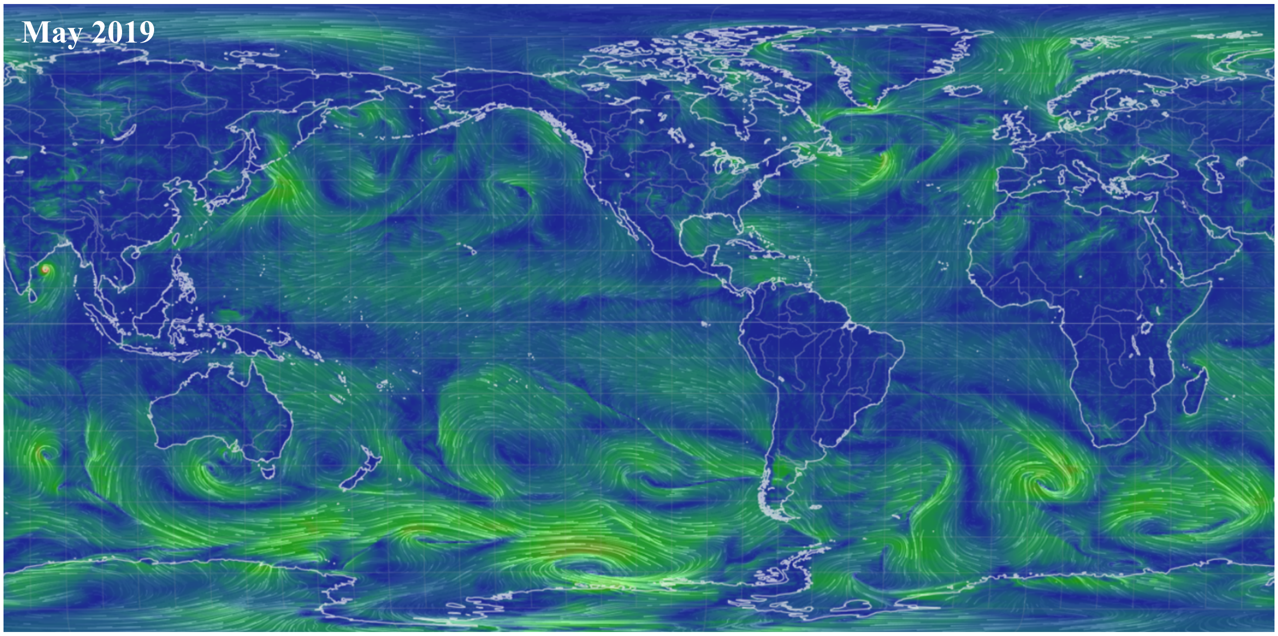
May 2019

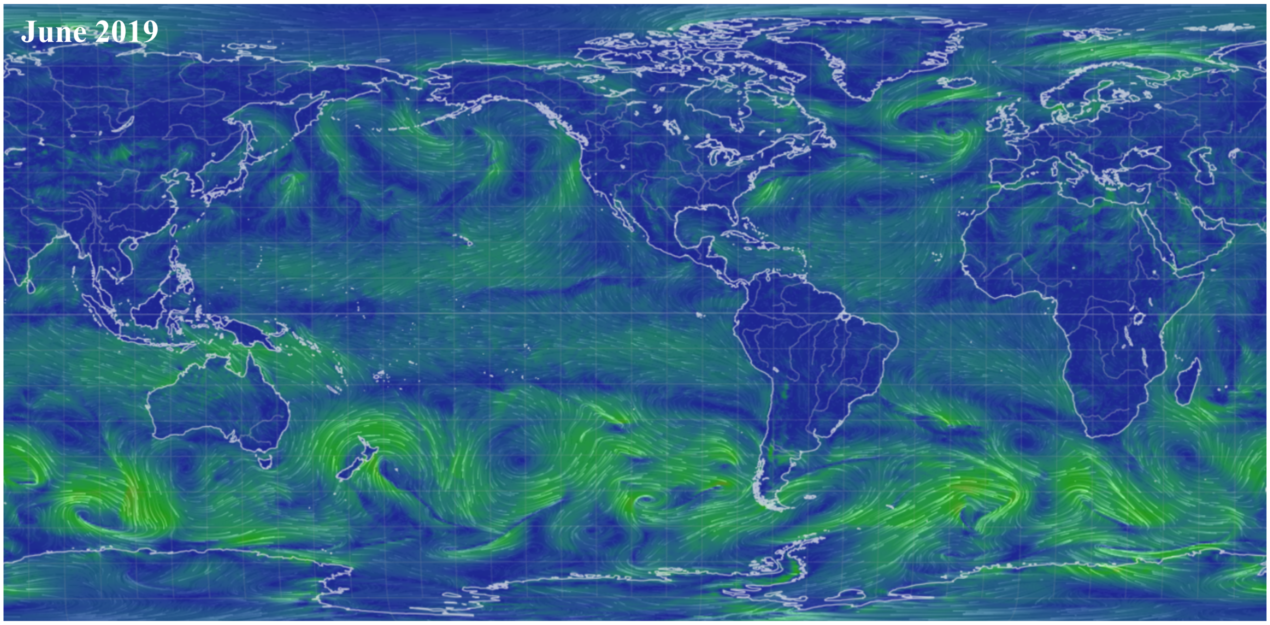
June 2019

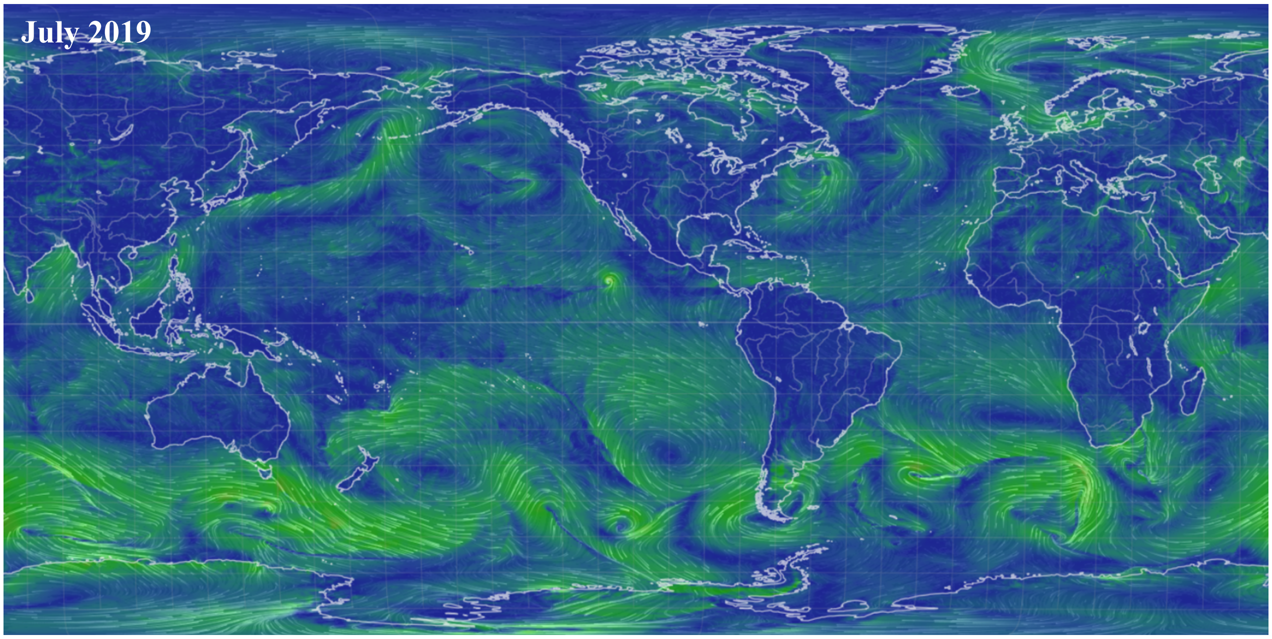
July 2019

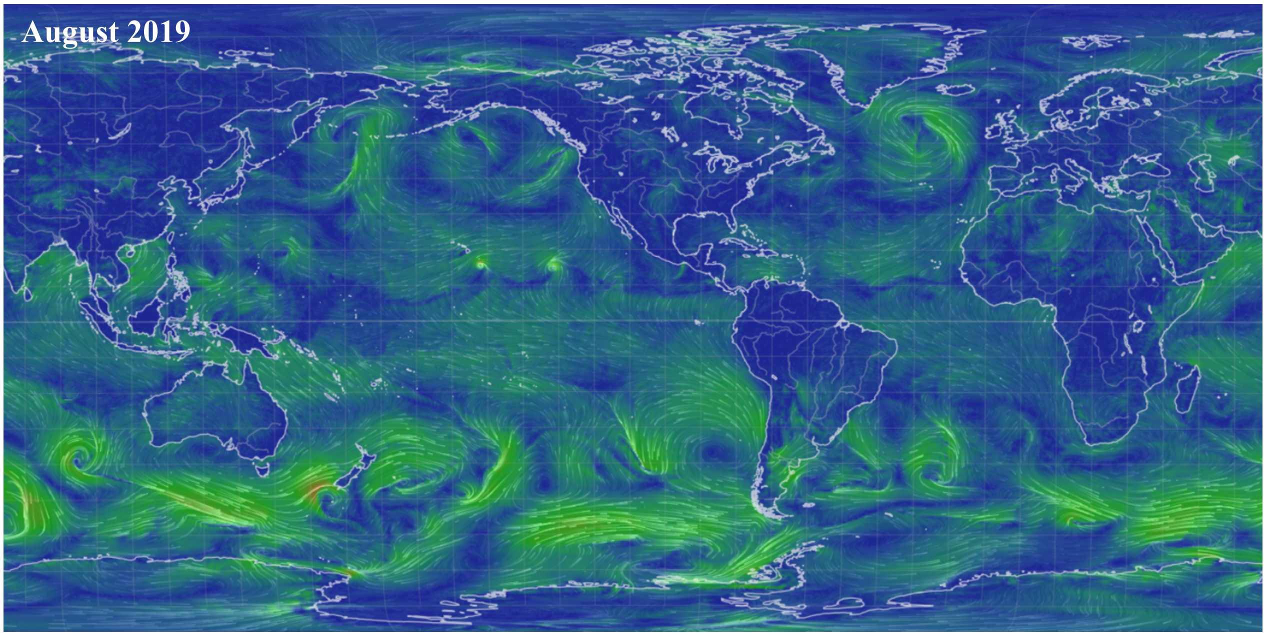
August 2019

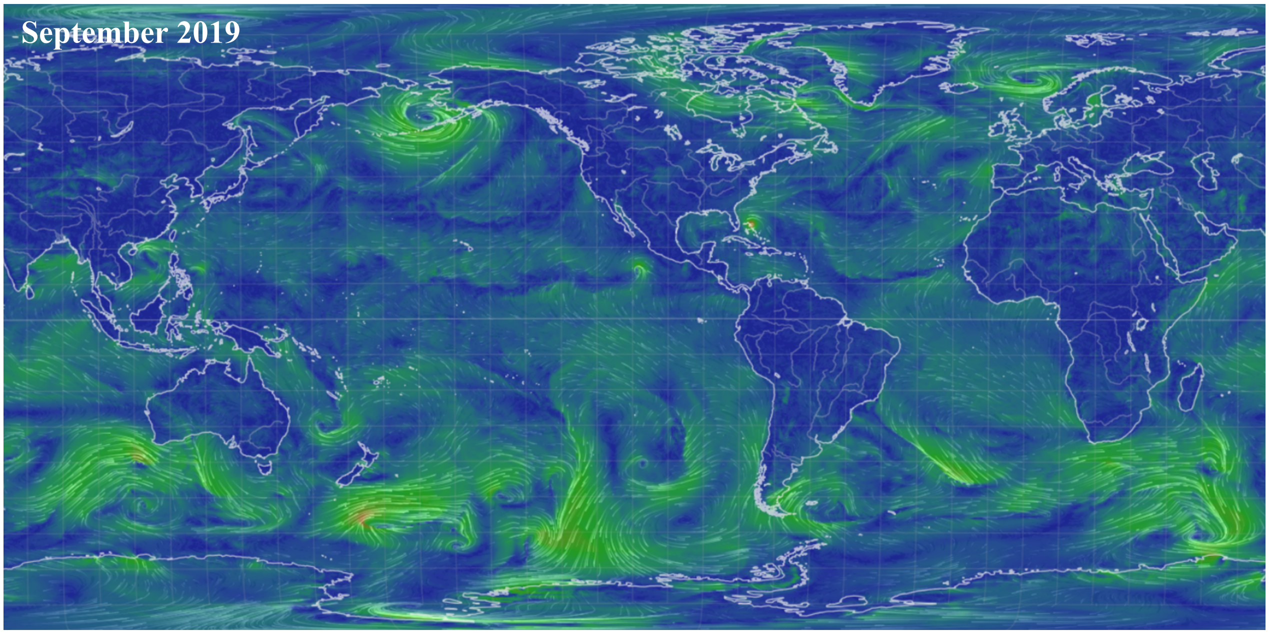
September 2019

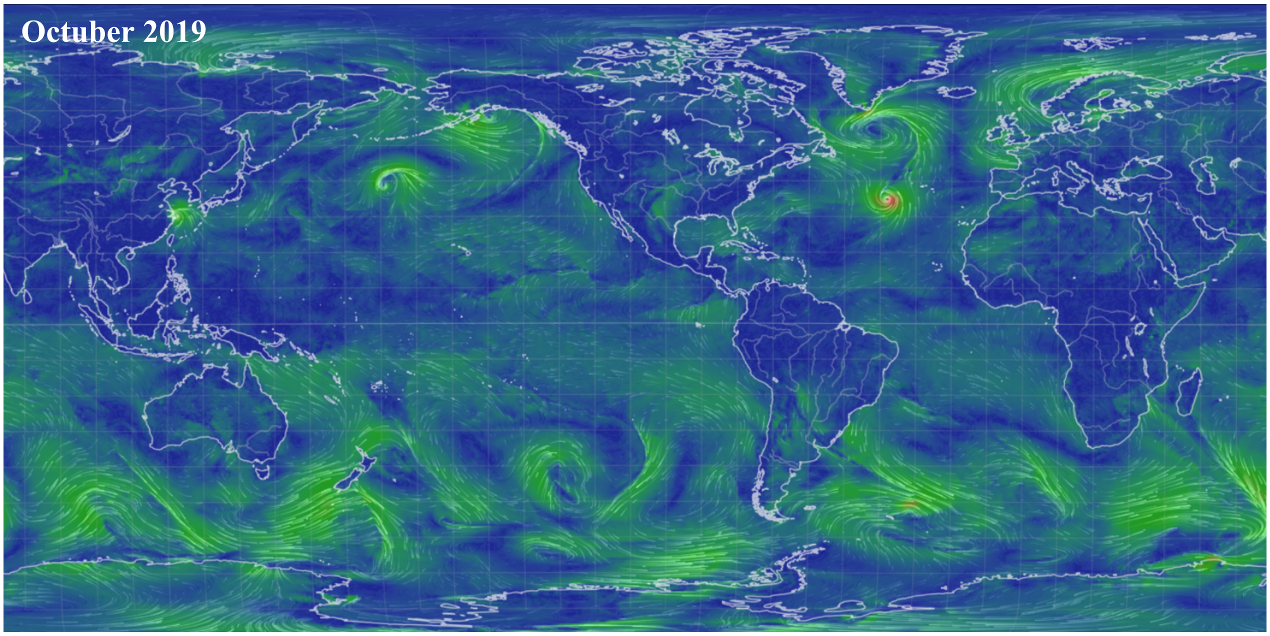
Octuber 2019

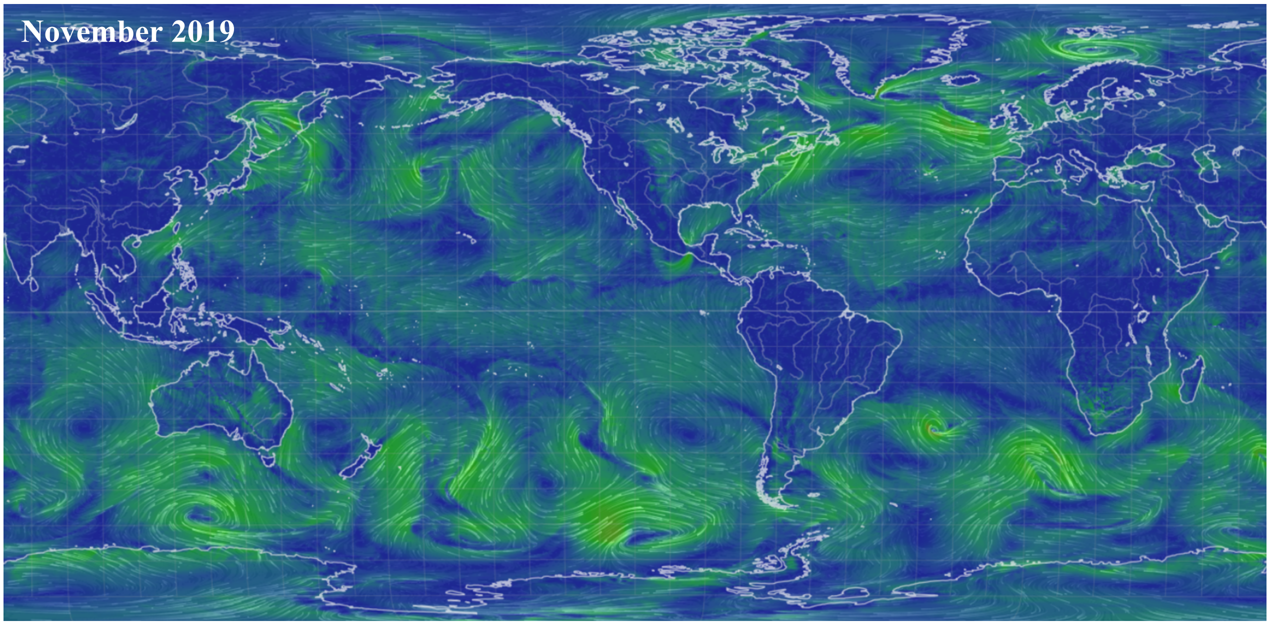
November 2019

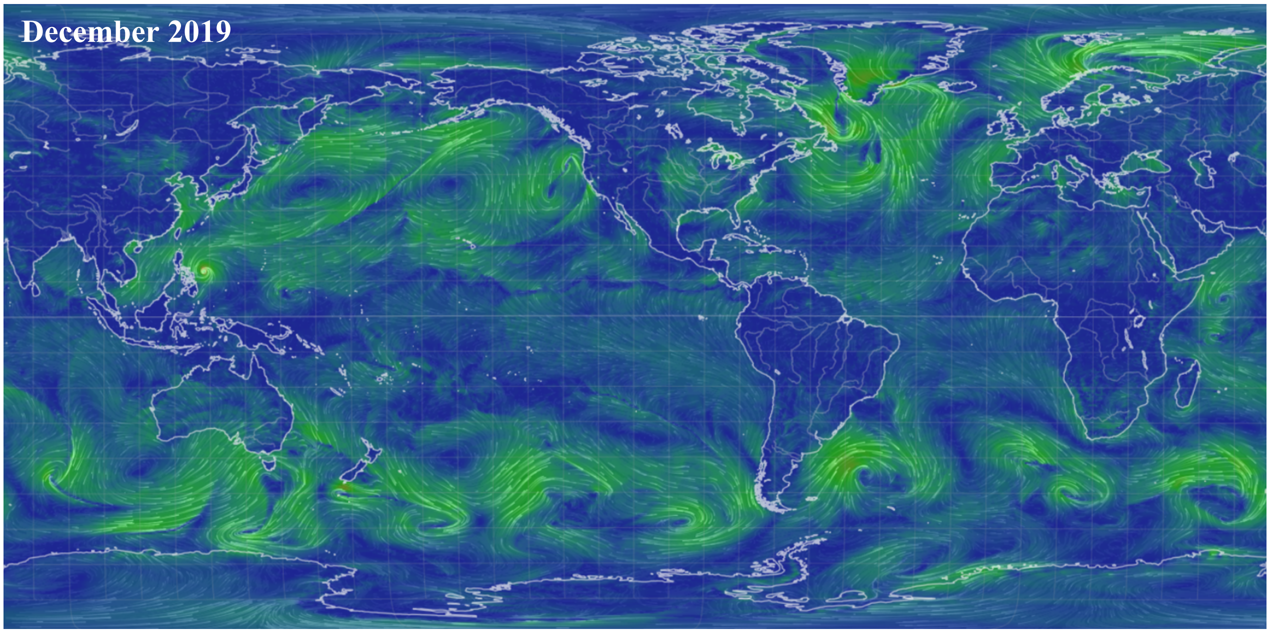
December 2019

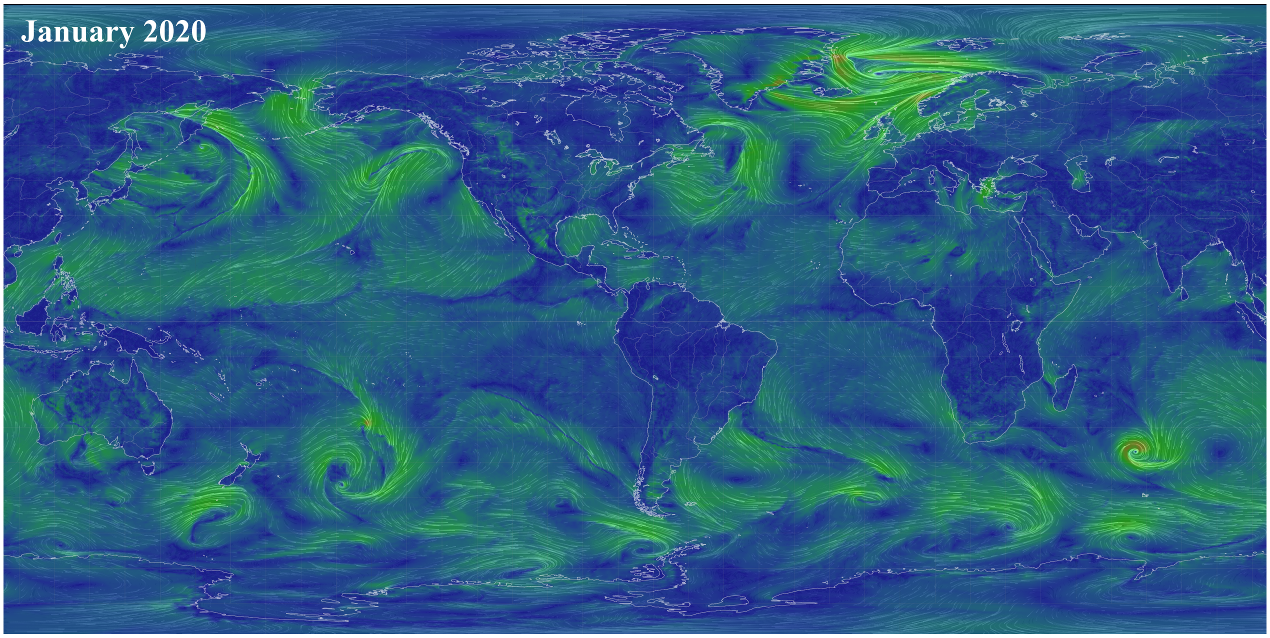
January 2020

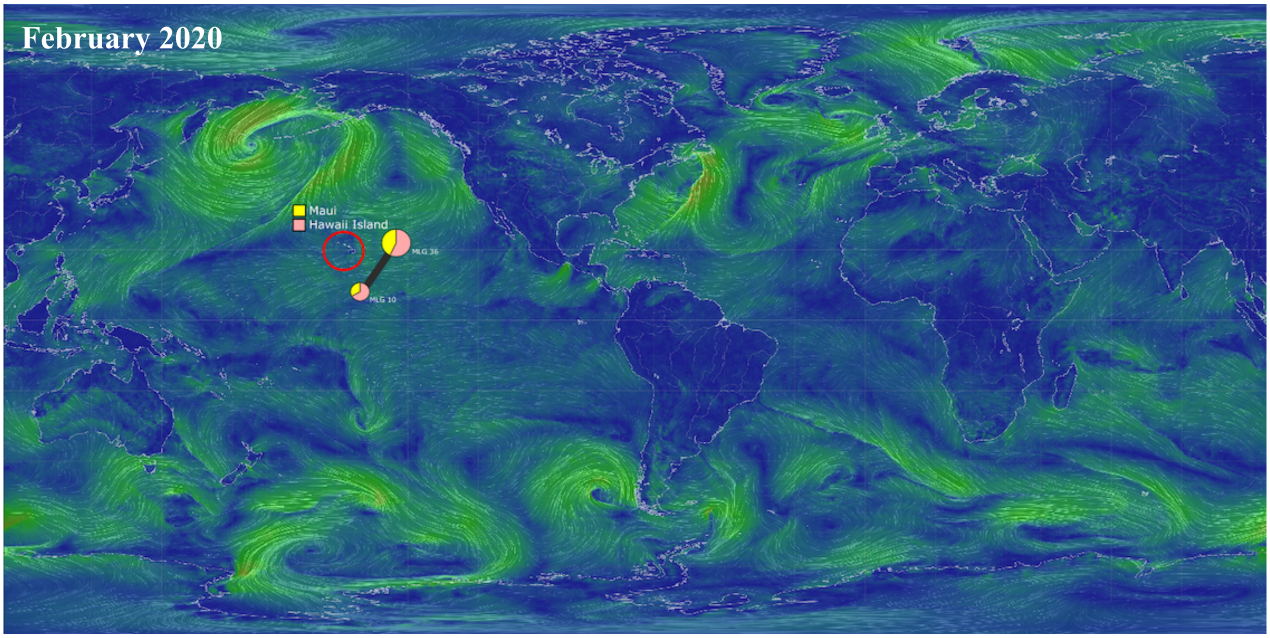

Figure S3. Global wind pattern during the first day each from February 2019 to October 2020. Air flow is denoted in green lines. For February (first detection of *Hemileia vastatrix*), the Hawaiian Islands are shown in a red circle. Genotypes mapping across Hawaii based in SSR.
